# Transcriptome Reprogramming of Symbiodiniaceae *Breviolum minutum* in Response to Casein Amino Acids Supplementation

**DOI:** 10.3389/fphys.2020.574654

**Published:** 2020-11-19

**Authors:** Andrea L. Kirk, Sophie Clowez, Fan Lin, Arthur R. Grossman, Tingting Xiang

**Affiliations:** ^1^Department of Biological Sciences, The University of North Carolina at Charlotte, Charlotte, NC, United States; ^2^Department of Plant Biology, Carnegie Institution for Science, Stanford, CA, United States; ^3^Brightseed Inc., San Francisco, CA, United States

**Keywords:** transcriptomics, topoisomerase, histone, DNA conformation, transport, phosphorylation, Symbiodiniaceae, symbiosis

## Abstract

Dinoflagellates in the family Symbiodiniaceae can live freely in ocean waters or form a symbiosis with a variety of cnidarians including corals, sea anemones, and jellyfish. Trophic plasticity of Symbiodiniaceae is critical to its ecological success as it moves between environments. However, the molecular mechanisms underlying these trophic shifts in Symbiodiniaceae are still largely unknown. Using *Breviolum minutum* strain SSB01 (designated SSB01) as a model, we showed that Symbiodiniaceae go through a physiological and transcriptome reprogramming when the alga is grown with the organic nitrogen containing nutrients in hydrolyzed casein, but not with inorganic nutrients. SSB01 grows at a much faster rate and maintains stable photosynthetic efficiency when supplemented with casein amino acids compared to only inorganic nutrients or seawater. These physiological changes are driven by massive transcriptome changes in SSB01 supplemented with casein amino acids. The levels of transcripts encoding proteins involved in altering DNA conformation such as DNA topoisomerases, histones, and chromosome structural components were all significantly changed. Functional enrichment analysis also revealed processes involved in translation, ion transport, generation of second messengers, and phosphorylation. The physiological and molecular changes that underlie *in vitro* trophic transitions in Symbiodiniaceae can serve as an orthogonal platform to further understand the factors that impact the Symbiodiniaceae lifestyle.

## Introduction

Endosymbiotic dinoflagellates from the family Symbiodiniaceae enter symbiosis with cnidarians, which include corals, sea anemones, and jellyfish (Davy et al., 2012; Fransolet et al., 2012; Bucher et al., 2016); this association is fundamental to the survival of coral-reef ecosystems. Cnidarian hosts benefit from the photosynthetically fixed carbon received from the alga (Muscatine and Porter, 1977; Burriesci et al., 2012), utilizing it to meet its energy requirements and enhance calcification rates (Pearse and Muscatine, 1971). The endosymbiotic alga gains access to inorganic nutrients from the host, establishes a stable location in the water column, and is protected from grazers (Davy et al., 2012). The breakdown of the symbiotic relationship between corals and its endosymbiotic alga, or “coral bleaching,” occurs under stress conditions, including elevated temperature and pollution of reef habitats (Hoegh-Guldberg, 1999; Weis and Allemand, 2009). Coral bleaching plays a major role in the global decline of reef communities (Hughes et al., 2017), and yet we still know little about the molecular mechanisms that govern the establishment, maintenance and breakdown of the symbiotic association (Davy et al., 2012).

Symbiodiniaceae and corals often must acclimate to diverse environments with different availabilities of nutrients (Leal et al., 2015; Fox et al., 2019; Morris et al., 2019; Conti-Jerpe et al., 2020). The trophic flexibility of the Symbiodiniaceae algae is essential for survival during both free-living and intracellular growth, especially when environmental conditions challenge growth and physiological processes in both the animal and alga (Xiang et al., 2018; Morris et al., 2019). Free-living Symbiodiniaceae in the ocean have been shown to directly take up nutrients from their surroundings (D’Elia et al., 1983; Brading et al., 2013), although the waters around coral reefs are typically oligotrophic (low-nutrient) (Cook and D’elia, 1987). Recent work has shown that some Symbiodiniaceae algae can thrive under different trophic conditions; autotrophy, heterotrophy, and mixotrophy (Xiang et al., 2013, 2018). For instance, two Symbiodiniaceae algae, both formerly considered clade E (classifications have recently been updated), that were cultured from environmental samples and from the tissues of the coral *Alveopora japonica*, were able to survive through the acquisition of fixed carbon by heterotrophic feeding (Jeong et al., 2012). Low levels of nutrients, such as nitrogen (N), arrest cell division and elicit transcriptional and physiological responses that help the organism cope with the limited nutrient availability (Dagenais-Bellefeuille and Morse, 2013; Jiang et al., 2014; Li et al., 2020; Xiang et al., 2020), while excess inorganic N could impair processes in the alga and host and weaken the symbiotic association (Morris et al., 2019).

We previously reported that the Symbiodiniaceae alga *Breviolum minutum* strain SSB01 (designated SSB01 throughout), formerly placed in clade B (Lajeunesse et al., 2012; Xiang et al., 2013; Lajeunesse et al., 2018), grew rapidly under mixotrophic conditions (minimal medium supplemented with organic nutrients in the light), with slower growth in the absence of organic nutrients (Xiang et al., 2013). SSB01 is one of the well-studied species with available genome and transcriptome resources (Shoguchi et al., 2013; Xiang et al., 2015; Parkinson et al., 2016). It readily forms symbiosis with cnidarian hosts (Xiang et al., 2013; Hambleton et al., 2014; Maor-Landaw et al., 2020) and has been shown to grow under various trophic conditions (Xiang et al., 2013, 2018). This ability to accommodate different nutrient resources (organic and inorganic) affords the alga trophic flexibility within its host where nutrient conditions may fluctuate (Xiang et al., 2013); for example, the dynamic changes in nutrient availability may reflect both the level of various nutrients in the environment and changes in the density of the algal population within the host tissue (Xiang et al., 2020). The cnidarian host may also feed and transport ingested organic nutrients, such as amino acids, lipids and fatty acids to the endosymbiont, potentially creating a mixotrophic interaction for the algae (Steen, 1986; Wang and Douglas, 1999; Imbs et al., 2014). However, the physiological responses and molecular mechanisms that guide metabolic acclimation to changes in the types and levels of available nutrients are not well understood.

In this study, to explore the physiological and molecular mechanisms that underlie trophic shifts in the Symbiodiniaceae, we analyzed the physiology and transcriptome profiles of axenic SSB01 cultured under various nutrient conditions: Artificial Sea Water (ASW), ASW supplemented with inorganic nutrients (IMK), and IMK supplemented with casein amino acids (CAS), which contains a variety of organic nutrients. Inclusion of CAS in IMK improved growth, allowed sustained photosynthetic function, and caused extensive transcriptome changes relative to cells maintained in either ASW or IMK media. Furthermore, there were only minor changes in the transcriptome of the algae grown in IMK compared to ASW. An understanding of the physiological and molecular features that underlie trophic transitions in the Symbiodiniaceae can provide insights into trophic changes associated with the growth of Symbiodiniaceae algae within their cnidarian hosts and help establish more general “rules” that govern symbiotic associations.

## Materials and Methods

### Strain and Growth Conditions

The clonal, axenic Symbiodiniaceae *B. minutum* (formerly *Symbiodinium minutum*) strain SSB01 (Xiang et al., 2013) was used throughout this study. Liquid cultures of SSB01 were grown either in ASW, ASW supplemented with 0.252 g L^–1^ of Daigo’s IMK medium for marine microalgae (Wako Pure Chemicals, Osaka, Japan) as recommended in the manufacturer’s instructions (IMK medium), or in IMK medium supplemented with 4 gL^–1^ casein hydrolysate (CAS; Affymetrix USB) (Xiang et al., 2013). Cultures were maintained at 27°C on a 12 h-light/12 h-dark cycle with an irradiance of ∼10 μmol photons m^–2^ s^–1^ provided by Philips ALTO II 25-W bulbs. To assess the effects of different nutrients on SSB01, approximately 2 × 10^7^ SSB01 cells from an IMK culture in log growth were collected by centrifugation at 100 *g* for 5 min in an Eppendorf 5810R centrifuge at room temperature. Cells were washed once with 50 mL autoclave-sterilized ASW and resuspended in 50 mL of either ASW medium, IMK medium or IMK + CAS medium in 250-mL flasks. For the recovery experiments, SSB01 cells were grown in ASW for 20 days. On day 20, the cells were pelleted by centrifugation at 100 *g* for 5 min at room temperature (RT) in an Eppendorf 5810R centrifuge and then resuspended in IMK + CAS and allowed to grow for an additional 20 days.

### Analysis of Photosynthetic Function

The cultures were prepared as described in Xiang et al. (2018, 2020) for measuring photosynthetic function. Maximum quantum yields of photosystem II (PSII) (calculated as *F*_v_/*F*_m_ = (*F*_m_–*F*_0_)/*F*_m_) were determined for cell cultures using a JTS-10 spectrophotometer (Bio-Logic) (Joliot and Delosme, 1974) after ∼10 min of dark adaptation.

### Growth Studies

SSB01 stock cultures were grown in IMK medium at 27°C on a 12 h-light/12 h-dark cycle with an irradiance of ∼10 μmol photons m^–2^ s^–1^. Approximately 6 × 10^6^ cells in log phase growth were pelleted by centrifugation at 100 *g* for 5 min at RT. Cells were washed twice with 20 mL sterile ASW medium, and resuspended in 30 mL of liquid ASW, IMK, or IMK + CAS media. SSB01 cells were quantified using a Countess^TM^ II Automated Cell Counter following the manufacturer’s instructions. Three biological replicates were performed for the growth experiments; they all yielded similar results.

### RNA-Seq Analyses

Cultured SSB01 cells grown in different nutrient conditions were prepared as described in “growth studies” (above). Approximately 5 × 10^7^ mid-log phase SSB01 cells grown in ASW, IMK, and IMK + CAS were collected by centrifugation and extracted with phenol/chloroform to prepare total RNA (Xiang et al., 2015; Xiang, 2018). RNA was prepared from three biological replicates for cells grown in IMK and IMK + CAS, and two biological replicates for cells grown in ASW. ASW cultures were transferred from IMK precultures and allowed to grow for 2 weeks before proceeding to RNA extraction. Approximately 1 μg of total RNA from each sample was used to construct libraries with the TruSeq RNA Sample Prep Kit (Illumina FC-122–1001) following the manufacturer’s instructions. The resulting libraries were sequenced on an Illumina HiSeq 2000 sequencer (2 × 101 bp) at the Stanford Center for Genomics and Personalized Medicine. All raw sequencing reads are available in the Sequence Read Archive^1^ with SRA accession numbers SRS5754975 (CAS samples) in the BioProject PRJNA591730 (Xiang et al., 2020), and SRS6837549 (IMK samples) and SRS6837550 (ASW samples) in the BioProject PRJNA639352. RNA-seq reads for populating symbiotic SSB01 samples (that were populating *Aiptasia* for 12 days and 30 days) were obtained from SRA, Project PRJNA261862 (Baumgarten et al., 2015). Differential expression analysis was performed as previously described (Xiang et al., 2020). Briefly, RNA-seq raw reads of each SSB01 sample grown in ASW, IMK, or CAS were aligned to the SSB01 transcriptome assembly Symb6 that we established previously (Xiang et al., 2015) [deposited at SRA, Project PRJNA591070^2^ using BWA (Li and Durbin, 2009)]. The number of reads aligned to transcripts with a cutoff mapping quality score of 30 was counted using SAMtools. Differential expression from different nutrient conditions was further analyzed using the DESeq2 Bioconductor package (Love et al., 2014), with the transcripts comparing IMK vs. ASW, and IMK + CAS vs. ASW. Expression levels were analyzed as transcripts per kilobase million (TPM) (Wagner et al., 2012). Differential expression of transcripts was called based on the cutoff of a false-discovery rate (Benjamini-Hochberg method) adjusted *p*-value of ≤0.001. GO-term enrichment was analyzed using the BiNGO plugin for Cytoscape (Maere et al., 2005).

### Amino Acid-Sequence Alignment

Amino acid sequence alignments for topoisomerases in SSB01 was conducted using MUSCLE (Edgar, 2004). 11 topoisomerase protein sequences predicted in symb6 and a total of 17 topoisomerase protein sequences from Arabidopsis (*Arabidopsis thaliana*; TOP1A, TOP1B, TOP2, TOP3A, TOP3B, TOP6A, TOP6B, TOP6BL, and GYRA), yeast (*Saccharomyces cerevisiae*, TOP1, TOP2, and TOP3), and human (*Homo sapiens*, TOP1, TOP2A, TOP2B, TOP3A, and TOP3B) were aligned. Protein sequences from the model systems were retrieved from the UniProt database with identifier IDs shown in Supplementary Table S1. The phylogenetic tree was constructed using the neighbor-joining method of Geneious tree builder in Geneious 9.1^3^.

## Results and Discussion

### IMK + CAS Medium Allows Faster Growth and Sustains Photosynthetic Function

To assess the impacts of inorganic and organic nutrients on the physiology of SSB01, we grew the cells in three different media with different nutrient compositions: (1) ASW; (2) IMK, which includes nitrate and ammonium; and (3) IMK + CAS. SSB01 cells were transferred from IMK medium to ASW, IMK, and IMK + CAS, respectively, and the growth characteristics and measurements of maximum quantum efficiency of PSII (*F*_v_/*F*_m_) were monitored.

**FIGURE 1:**
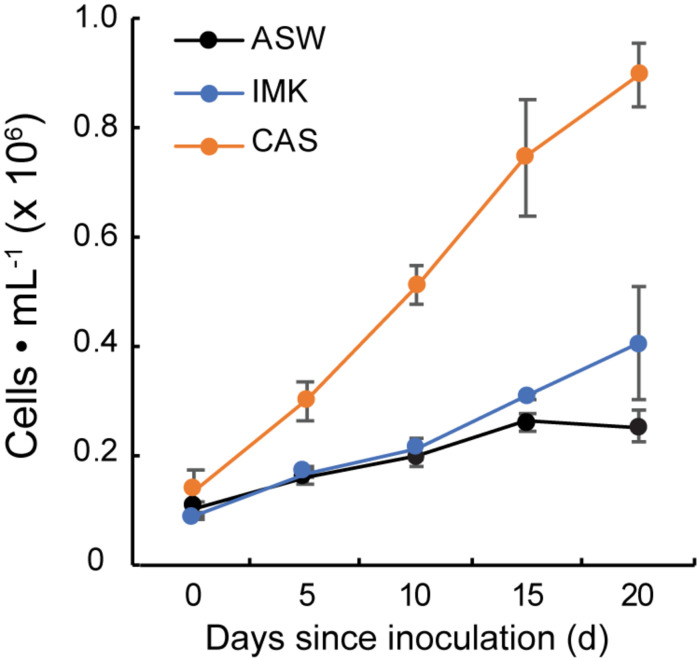
Growth of SSB01 cells in ASW, IMK, and IMK + CAS. Growth of SSB01 was assessed in ASW (black line), IMK (blue line), and IMK + CAS (CAS, orange line) medium for 20 days. Error bars show standard errors derived from three replicate experiments.

**FIGURE 2:**
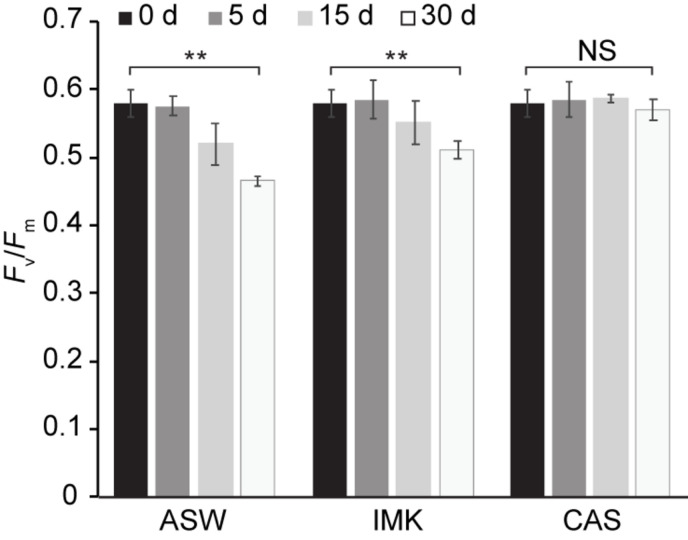
Stability of PSII efficiency for SSB01 cells grown in ASW, IMK, and IMK + CAS (CAS). The *F*_v_/*F*_m_ was measured (see “Materials and Methods”) at 0, 5, 15, and 30 days when SSB01 cells were grown in ASW, IMK, and IMK + CAS, respectively. *p*-values (two sided *t*-test) for the significance of the 0 vs. 30 days differences are indicated (**p-value < 0.01, NS, not significant).

**FIGURE 3:**
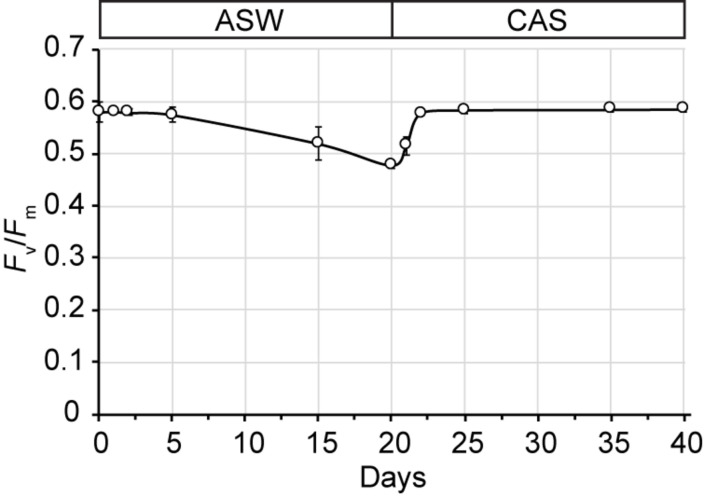
Recovery of *F*_v_/*F*_m_ after transfer of ASW-grown SSB01 cells to IMK + CAS medium (CAS). Cells were grown in seawater for 20 days and then transferred to IMK + CAS medium; the *F*_v_/*F*_m_ was measured for cells before and after the transfer. Error bars show standard errors derived from three replicate experiments.

Growth of SSB01 was significantly more rapid in IMK + CAS (*p*-value is 3.05E-05 based on two-sided *t*-test) and cells were able to reach much higher densities compared to ASW (during the 20 days of growth), while the difference between growth rates in IMK and ASW was small (*p*-value is 0.06 based on two-sided *t*-test) (Figure 1). The *F*_v_/*F*_m_ remained stable over a growth period of 30 days in IMK + CAS but exhibited a significant decrease when the cells were grown in ASW (*p*-value = 0.004, two-sided *t*-test) or IMK (*p*-value = 0.006, two-sided *t*-test) for the same period of time (Figure 2). Interestingly, the decline in *F*_v_/*F*_m_ was fully reversed in 2 days following supplementation of ASW-grown SSB01 cells with CAS; it remained stable for the additional 20 days of the experiment (Figure 3).

### Extensive Transcriptome Changes in Response to IMK + CAS

**FIGURE 4:**
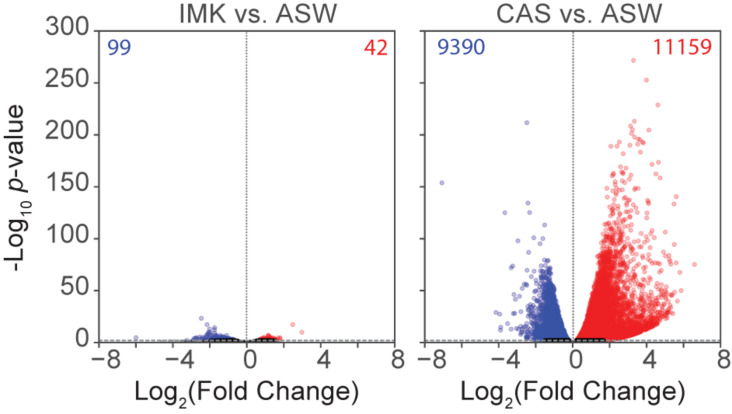
Expression in SSB01 in IMK + CAS or IMK compared to ASW. Volcano plot of relative abundances of individual transcripts in IMK vs. ASW and IMK + CAS vs. ASW. *x*-axis, fold-changes; *y*-axis, adjusted *p*-values based on Benjamini-Hochberg correction; red dots indicate transcripts more abundant in IMK (left) or in IMK + CAS (right). Blue dots indicate transcripts more abundant in ASW. Both axes use log scales. The horizontal line indicates adjusted *p*-values = 0.001, the cutoff used for considering differences to be significant.

To determine the changes in gene expression potentially associated with the physiological observations and to gain new insights into the acclimation of the cells to different trophic conditions, RNA-Seq was conducted for SSB01 grown in ASW, IMK, and IMK + CAS, with comparisons of transcript abundances of IMK + CAS relative to ASW, and IMK relative to ASW. Based on a cutoff of adjusted *p*-values (based on Benjamini-Hochberg correction) ≤0.001, we identified 20,549 transcripts (approximately 34.4%) that were differentially expressed when comparing cells grown in IMK + CAS relative to ASW (Figure 4). In contrast, only 141 transcripts (approximately 0.2%) were scored as differentially expressed when comparing SSB01 cells grown in IMK relative to ASW (Figure 4).

**FIGURE 5:**
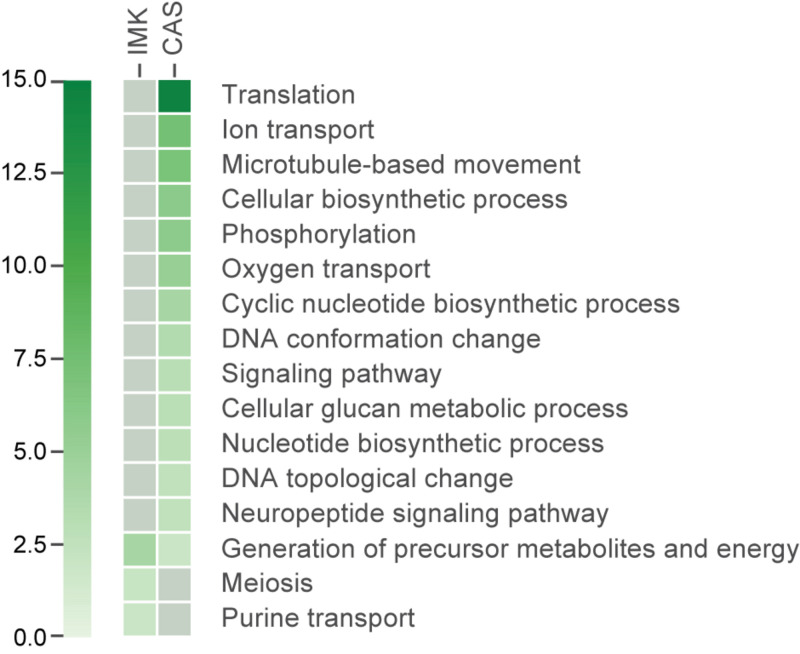
The GO categories enriched (based on adjusted hypergeometric *p*-value < 0.1) in the transcripts showing significant differential expression. Heatmap showing the *p*-value for the enrichment of each category. Gray colored boxes indicate not significant.

We applied a Gene Ontology (GO) enrichment analysis and further analyzed the functional categories of the differentially expressed genes. GO terms for the proteins encoded by the transcripts enriched in IMK + CAS relative to ASW were represented by a wide variety of processes including “translation,” “DNA conformation change” (“DNA topological change” in particular), “ion transport,” cytoskeleton such as “microtubule-based movement,” “phosphorylation” (mostly “protein amino acid phosphorylation”), “oxygen transport” which is fundamental to aerobic respiration, “nucleotide biosynthetic process,” “cyclic nucleotide biosynthetic process,” “signaling pathway,” “neuropeptide signaling pathway,” and “cellular glucan metabolic process.” By contrast, few GO terms were enriched for the proteins encoded by transcripts expressed in IMK relative to ASW, but those terms that were slightly enriched include “generation of precursor metabolites and energy,” “purine transport,” and “meiosis” (Figure 5).

### CAS May Modulate Translation and Protein Synthesis

The greatest number of genes that showed differential regulation in IMK + CAS relative to ASW medium encode proteins associated with translation. The transcript (s6_33548) encoding the General Control Non-derepressible 2 (GCN2) exhibited an ∼18-fold increase in IMK + CAS. GCN2 is a serine/threonine-protein kinase that acts as an amino acid sensor in *S. cerevisiae* and can affect expression of genes encoding enzymes involved in amino acid synthesis (Castilho et al., 2014). Symbiodiniaceae GCN2 may perform similar functions in amino-acid sensing. Interestingly, the expression levels of *GCN2* in symbiotic SSB01 increased gradually when the algal symbiont population increased (Supplementary Figure S1). This data suggests that sensing and controlling the biosynthesis of amino acids in the symbiont may be integral to host-symbiont interactions as the symbiont populates the host.

Elongation factor thermo unstable (EF-Tu) is an essential component of translation that places the aminoacyl-tRNA complex at the A-site of the ribosome (Rodnina et al., 2005). The transcript encoding EF-Tu (s6_1943) increased in abundance by more than 3-fold in IMK + CAS. The transcript encoding valine tRNA ligase also showed a >2-fold change in abundance. In addition, expression of 15 transcripts involved in translation initiation were significantly changed in IMK + CAS. For example, the abundance of the transcript (s6_51857) encoding the eukaryotic translation initiation factor 3 subunit B protein (eIF-3B) increased by almost 3-fold in IMK + CAS. This translation initiation factor is an RNA-binding component of the eukaryotic translation initiation factor 3 (eIF-3) complex, which specifically initiates translation of a subset of mRNAs involved in cell proliferation (Lee et al., 2015). In contrast, the level of the transcript (s6_38195) encoding translation initiation factor 5A decreased by almost 3 fold in IMK + CAS (Supplementary Table S3). This translation initiation factor was recently reported to be involved in polypeptide elongation rather than initiation (Gregio et al., 2009; Saini et al., 2009) and was shown to stimulate protein synthesis in *S. cerevisiae* (Henderson and Hershey, 2011). The precise meaning of these results is unclear, although measuring the intracellular levels of amino acids in both ASW and IMK + CAS grown cells may provide insights into potential feedback signals that might be involved in modulating protein homeostasis through the controlled expression of genes related to translation and protein synthesis.

### Changes in Transcripts Encoding Transporters Associated With Limiting Nutrient Availability

Many changes in expression were observed for transcripts related to ion transporters, with 402 transcripts differentially expressed when SSB01 cells were grown in IMK + CAS (Supplementary Table S6). Of those differentially expressed in IMK + CAS, 279 showed elevated transcript accumulation while 123 showed reduced transcript levels (Supplementary Table S6). Transcripts encoding voltage-gated sodium channel and voltage-gated ion channel superfamily proteins were some of the most highly expressed in IMK + CAS. For example, the level of the transcript (s6_2946) encoding a voltage-gated sodium channel increased by more than 7-fold. Additionally, four transcripts (s6_9034, s6_3890, s6_2386, s6_1118) encoding chloride channel proteins increased in abundances by more than 2-fold. Voltage-gated ion channels are transmembrane proteins that function in action potential generation in animal, plant, and algal cells (Ward et al., 2009). Levels of transcripts encoding calcium channels were also elevated in IMK + CAS, with 15 of these transcripts exhibiting ∼2-fold increase in abundance relative to ASW (Supplementary Table S6). Calcium is an essential micronutrients and calcium signaling is critical for the cells to decode internal and external stimuli, and transduce them into changes in gene expression that modulates physiological processes (Demidchik et al., 2018). In addition, many transcripts involved in nitrogen transport including ammonium transporters and nitrate transporters were significantly changed in IMK + CAS. 20 transcripts encoding ammonium transporters were preferentially expressed in ASW while three were elevated (s6_7783, s6_5492, s6_32551) in IMK + CAS. However, only one transcript encoding an ammonium transporter (s6_51578), and one transcript involved in purine transport (s6_1521) were differentially expressed in IMK relative to ASW. The abundance of the transcript s6_51578 decreased >4-fold in both IMK and IMK + CAS relative to ASW. Similarly, we found seven transcripts encoding nitrate transporters that were expressed at higher levels in ASW (Supplementary Table S6). Symbiodiniaceae increases the level of transcripts encoding most transporters associated with the uptake of nitrogen compounds when the cells become limited for nitrogen (Li et al., 2020; Xiang et al., 2020). Previous studies have also shown that members of the former clade B Symbiodiniaceae displayed decreased growth rates under nitrogen-deprivation (Jiang et al., 2014). The elevation of transcripts encoding ammonium and nitrate transporters observed for cells maintained on ASW medium suggests that SSB01 is experiencing nitrogen limitation, which is consistent with its slow growth (Figure 1) and the decreased maximum efficiency of photosystem II (Figure 2) in ASW medium. The presence of organic nutrients along with appropriate amounts of ammonium and nitrate were suggested to increase the health of the holobiont (Morris et al., 2019). The transcriptome responses observed for SSB01 cells grown in IMK + CAS compared to ASW medium also suggests that ASW-grown cells are experiencing nitrogen deprivation and that the amino acids of CAS may provide the alga with a sufficient supply of nitrogen; this also suggests that when necessary, organic nutrients, including amino acids, may be provided by the host to the symbiont (Steen, 1986; Wang and Douglas, 1999; Imbs et al., 2014).

### IMK + CAS May Elevate the Synthesis of Cyclic Nucleotides

The cyclic nucleotides cAMP and cGMP are important second messengers that modulate many fundamental cellular processes including metabolism, development and differentiation, cell proliferation, and cell survival under adverse conditions (Scheib et al., 2018). Transcripts encoding adenylyl cyclase and guanylate cyclases, enzymes that synthesize cAMP and cGMP (Steer, 1975; Potter, 2011), respectively, were differentially expressed in SSB01 cells grown in IMK + CAS relative to ASW and IMK (Supplementary Table S4). Of the 21 transcripts encoding adenylyl and guanylate cyclases, 19 increased while only two decreased in IMK + CAS relative to ASW or IMK. The potential increase in the synthesis of adenylyl and guanylate cyclases in IMK + CAS raises the possibility that organic nutrients/amino acids may trigger the production of second messengers such as cGMP and cAMP (Carucci et al., 2000), which in turn might regulate aspects of Symbiodiniaceae cell growth, proliferation and/or might enable the cells to better cope with suboptimal environmental conditions. Two decades ago cAMP was shown to regulate cell cycle progression and growth of the dinoflagellate *Crypthecodinium cohnii* (Lam et al., 2001).

### Massive Changes in Abundances of Transcripts for Enzymes Involved in Protein Phosphorylation

Dinoflagellates appear to have permanently condensed chromosomes and transcriptional control may be limited (e.g., transcript levels rarely become very high during acclimation processes), suggesting that post-translational modifications play important roles in dinoflagellates responses to environmental change (Leggat et al., 2007; Krueger et al., 2015; Rosic et al., 2015; Xiang et al., 2015; Gierz et al., 2016). Quantification of transcripts encoding enzymes involved in protein phosphorylation strongly suggest that phosphorylation may be modulating many cellular processes in response to different nutrient conditions. There are 64 transcripts encoding calcium-dependent protein kinases that are differentially expression in IMK + CAS relative to IMK (and ASW) (Supplementary Table S5); 50 of these differentially expressed transcripts were elevated while 14 were diminished in IMK + CAS. For example, a transcript encoding a calcium dependent protein kinase (s6_33927) increased by almost 10-fold in IMK + CAS relative to ASW. Furthermore, 17 transcripts related to cGMP-dependent protein kinases increased in IMK + CAS while only one decreased. The largest change in abundance for cGMP-dependent kinases was for cGMP-dependent protein kinase isozyme 1 (s6_7176), which exhibited a greater than 16-fold elevation in IMK + CAS relative to ASW (Supplementary Table S5). The increase observed for transcripts related to cGMP-dependent protein kinases is congruent with changes in the levels of transcripts associated with cyclic nucleotide metabolism. Overall, our results indicate that growth of SSB01 in a source of organic nitrogen elicits an increase in the synthesis of adenylyl and guanylate cyclases and cyclic nucleotide dependent kinases, which likely promotes signal transductions through cGMP and cAMP second messengers.

### Changes in the Levels of Transcripts for DNA Topoisomerases and Histones Suggest That the Chromosomes Experience Nutrient Driven Changes in DNA Conformation

Some notable differences in cells maintained in IMK + CAS relative to IMK or ASW involves transcripts encoding proteins associated with DNA topography. Dinoflagellate chromosomes are permanently condensed and maintain a liquid crystalline state that does not appear to rely on histones (Wisecaver and Hackett, 2011). Differences in mechanisms controlling DNA replication and transcription in dinoflagellates may occur as a consequence of their permanently condensed chromosomes and the absence of nucleosomes or the presence of divergent nucleosomes. It has been suggested that extrachromosomal loops protruding from the condensed chromatin structure may allow access of the DNA to the cell’s transcriptional machinery (Wisecaver and Hackett, 2011). However, analysis of the 3-dimensional organization of the *B. minutum* genome revealed large topological domains demarcated by convergent gene array boundaries [“dinoTADs,” topologically associating domains (TADs) in dinoflagellates], possibly formed as a consequence of transcription-induced supercoiling (Marinov et al., 2020).

Many transcripts encoding DNA topoisomerases were elevated in IMK + CAS relative to ASW medium. We identified 22 transcripts encoding topoisomerases I, II, and III in SSB01. These enzymes change the topology of DNA by overwinding or underwinding the polynucleotide strands and have been implicated in critical cellular functions including DNA replication, DNA repair, and transcription (Levin et al., 1993; Champoux, 2001). Topoisomerase I functions in DNA replication and transcription by creating a single-strand break that allows relief of strain caused by DNA supercoiling (Champoux, 2001). Type II topoisomerases, which can form dimers with Type III topoisomerases, similarly relieve supercoiling strain through the generation of double-stranded DNA breaks (Hartung et al., 2008; Ahmad et al., 2016). The atypical type II topoisomerase, DNA gyrase, is essential for negative supercoiling during replication and transcription (Kampranis and Maxwell, 1996).

**FIGURE 6:**
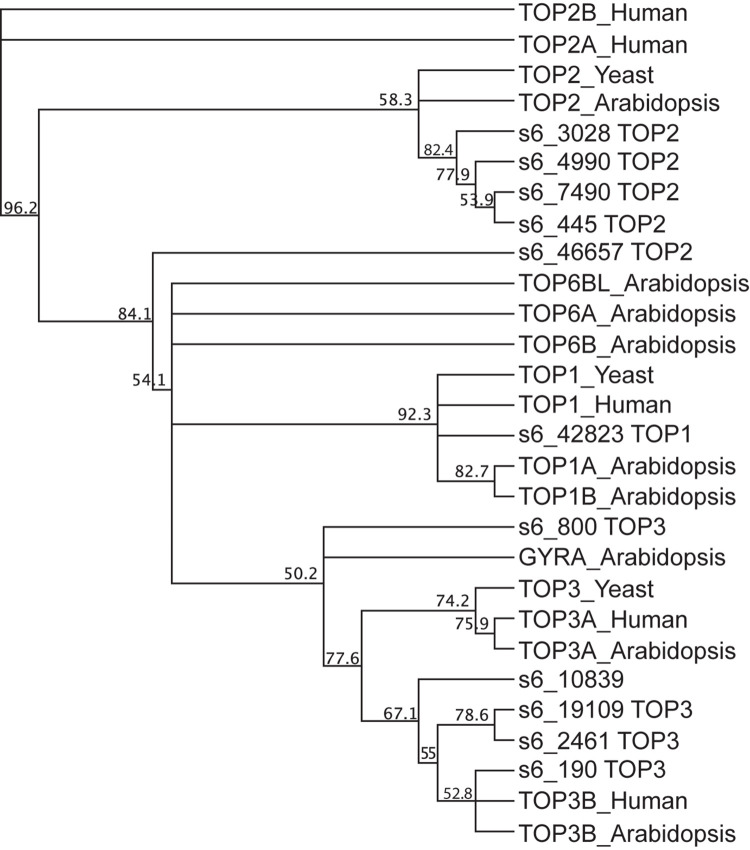
Phylogenetic analysis of DNA topoisomerase sequences in SSB01. Protein polypeptide sequences of 11 DNA topoisomerases in SSB01, 9 in Arabidopsis (*Arabidopsis thaliana*; TOP1A, TOP1B, TOP2, TOP3A, TOP3B, TOP6A, TOP6B, TOP6BL, and GYRA), 3 in yeast (*S. cerevisiae*, TOP1, TOP2, and TOP3), and 5 in human (*Homo sapiens*, TOP1, TOP2A, TOP2B, TOP3A, and TOP3B) were aligned using the MUSCLE program. The phylogenetic tree was built using the neighbor-joining method by Geneious tree builder. The numbers on the nodes indicate the percentage of bootstrap *p*-values obtained from 1,000 replicates.

The transcripts for 11 topoisomerases, marked with an asterisk in Supplementary Table S2, had significantly increased mRNA abundances in IMK + CAS relative to ASW (Supplementary Table S2). Of the 11 topoisomerase transcripts, one was type I, five were type II, and five were type III (Figure 6). The transcript for the DNA gyrase (s6_445) increased by more than 3-fold in IMK + CAS and also showed increased levels when symbiotic SSB01 algae populate the *Aiptasia* host (Supplementary Figure S1). Elevated transcripts for five Type II topoisomerases in *B. minutum* suggest that they may form a complex and participate in unwinding of DNA that relieves topological stresses and allows access of the genome to transcription factors.

Topoisomerases have been detected in various dinoflagellates (Mínguez et al., 1994; Mak et al., 2005) and it was suggested that *C. cohnii* type II topoisomerase unwinds condensed chromosomes of the G1 phase of the cell cycle for transcription (Mak et al., 2006). Our results also raise the possibility that these topoisomerases may be important for exposing regions of the chromosomal DNA during replication and transcription in the Symbiodiniaceae.

Previously it was thought that dinoflagellate histones play limited roles in DNA packaging, supercoiling and transcriptional changes, even though both conserved and divergent histones and the histone code were found in these organisms, including in *B. minutum*, through genomics analysis (Marinov and Lynch, 2015). Symbiodiniaceae possess transcripts for all core histones, but these histones do not appear to play major roles in the organization and packaging of nuclear DNA (Wisecaver and Hackett, 2011; Gornik et al., 2012). We identified several transcripts encoding core histones and histone protein variants that were differentially expressed when SSB01 was grown on IMK + CAS. Interestingly, the level of the transcript encoding a protein similar to the H3-like centromeric protein A (CENP-A, s6_36342) increased by more than 3-fold. Transcripts encoding Histone H3 (s6_4125 and s6_16949) and Histone H2A (s6_34360) were modestly downregulated. In addition, the expression levels of the transcript for CENP-A increased when SSB01 algae were populating the *Aiptasia* host (Supplementary Figure S1). CENP-A, a histone H3 variant, confers epigenetic identity to centromeres and promotes the assembly of kinetochores, chromosome segregation, and cell division (Black et al., 2007; McKinley and Cheeseman, 2016). Tight regulation of CENP-A is critical for proper centromere assembly. It is possible that histones and histone variants could play similar roles in Symbiodiniaceae in modulating chromatin structures and accessibility during cell proliferation. CENP-A has also been shown to prevent the binding of H1 and modify the wrapping of DNA in the nucleosome (Roulland et al., 2016). The elevation of the CENP-A transcripts and the decline of the H3 transcripts in SSB01 cells grown in IMK + CAS may allow for DNA to become more accessible for replication and transcription. These changes may reprogram cell metabolism with respect to its nutrient status.

We also observed massive changes in the levels of transcripts encoding motor proteins. Kinesins are microtubule-bound molecular motors that hydrolyze ATP to perform a range of functions, including the transport of organelles, modulation of cellular organization, mitosis, and signal transduction (Hirokawa et al., 2009; Marx et al., 2009). Many transcripts (53) encoding kinesin family proteins were differentially expressed in SSB01 cells grown in IMK + CAS (Supplementary Table S7). The most significantly upregulated transcript encoding a chromosome-associated kinesin (s6_51770) showed more than 6-fold elevation in IMK + CAS relative to ASW. These results suggest that the nutrient status of the medium could significantly impact the levels and functions of microtubule bound motors which may play essential roles in cell division and cellular organization and would be a critical point for control during growth *in hospite*.

Interestingly, the level of the transcript for nucleosome assembly protein-1 (NAP1, s6_2729) is also elevated in IMK + CAS. NAP1 functions in shuttling histones into the nucleus, assembling nucleosomes, and promoting chromatin fluidity, thereby regulating transcription. The loss of NAP1 in yeast resulted in prolonged delays in mitosis, and it is hypothesized that the stimulation of transcription factor binding by NAP1 could result in greater DNA accessibility (Park and Luger, 2006).

Structural maintenance of chromosomes (SMC) proteins are part of a large family of ATPases that form dimers at the core of the condensin complexes (Löwe et al., 2001; Hirano, 2002). As key organizers of chromosome architecture, SMC proteins play major roles in higher-order chromosome organization and dynamics, including chromosome condensation, cohesion of sister chromatids, DNA repair, and gene expression (Harvey et al., 2002). The levels of transcripts encoding SMC1-like protein (s6_5945) and SMC2 (s6_3357) increased in IMK + CAS, whereas another transcript encoding SMC1 (s6_638) decreased in abundance (Supplementary Table S2). In addition, the transcript encoding condensin complex subunit 1 (s6_5055), which is a regulatory subunit of the condensin complex, increased in abundance by almost 3-fold in IMK + CAS. Together, our results raise the possibility that the supplementation of IMK medium with CAS impacts Symbiodiniaceae chromosome architecture through the core histone proteins, histone chaperones, topoisomerases, and SMC ATPases.

## Conclusion

The study presented here provides insights into the responses of Symbiodiniaceae to organic and inorganic nutrients. When IMK medium is supplemented with CAS, a mixture of organic nitrogen containing compounds (mostly amino acids), SSB01 cells grow faster and maintain their photosynthetic apparatus; maximum quantum yield of photosystem II was diminished in cells cultivated for long periods in IMK or ASW. Analysis of differentially expressed transcripts showed that SSB01 grown in ASW and IMK showed few differences in transcript accumulation. In contrast, SSB01 cells grown in IMK + CAS exhibited significant differences in the abundances of transcripts that encode proteins associated with ion transport, translation, cyclic nucleotide biosynthesis, phosphorylation, and DNA conformation. Many of the changes appear to be related to rapid growth and cell division which may be coupled to the nutrient status of the cells. ASW is not conducive to rapid algal growth and may not provide the cells with adequate nutrients, as indicated by activation of nutrient scavenging pathways. Experiencing a scarcity of nutrients may also drive Symbiodiniaceae into establishing a symbiotic association with a cnidarian host, where nutrients may be more readily available and also play a major role in regulating host-symbiont interactions. More detailed physiological and molecular analyses of Symbiodiniaceae algae exposed to various conditions in culture may define the impact of light, temperature and nutrients (e.g., nitrogen, fixed carbon, phosphate) on growth and photosynthesis, suggest metabolic pathways associated with stress conditions and the different trophic life-styles (e.g., autotrophic, mixotrophic, heterotrophic), and provide a detailed picture of the ways in which these algae accommodate environmental change. Ultimately, by comparing transcriptional responses observed in culture with those associated with algae growing *in hospite* may help elucidate the *in hospite* conditions experienced by the endosymbiotic alga and identify key genes that are specifically involved in symbiosis. One important example of this is the elevation in levels of transcripts encoding GCN2 protein kinases, which may act as an amino acid/organic nitrogen sensor in SSB01, similar to its function in *S. cerevisiae* (Castilho et al., 2014).

## Data Availability Statement

The raw RNA-Seq reads datasets for this study can be found in the NCBI SRA with the accession number PRJNA639352.

## Author Contributions

TX conceived and planned the research. TX, SC, AK, FL, and AG analyzed the data. SC measured photosynthetic activities. AK, TX, and AG wrote the manuscript, with contributions from all the authors. All authors contributed to the article and approved the submitted version.

## Conflict of Interest

FL was employed by the company Brightseed Inc. The remaining authors declare that the research was conducted in the absence of any commercial or financial relationships that could be construed as a potential conflict of interest.

## Footnotes

1 http://www.ncbi.nlm.nih.gov/sra

2 https://www.ncbi.nlm.nih.gov/bioproject/PRJNA591070

3 https://www.geneious.com

## Supplementary Material

Supplementary Figure 1 SSB01 transcripts for GCN2, TOP2, and CENP-A under different culturing and symbiotic conditions. Transcript levels for General Control Nonderepressible 2 (GCN2, s6_33548), topoisomerase II (TOP2, s6_445) and histone h3-like centromeric protein a-like (CENP-A, s6_36342) expressed as transcripts per kilobase million(TPM) from *in vitro* cultures (ASW, IMK and CAS) and populating symbiotic SSB01 (12 d, 30 d, and steady state) inthe Aiptasia host. Results of symbiotic SSB01 were obtained by analyzing the data from Baumgarten et al., 2015; andXiang et al., 2020. Shown are means ± SDs from at least three biological replicates.

Supplementary Table 1 UniProt IDs of protein sequences used for amino acid-sequence alignment and phylogenetic tree.

Supplementary Table 2 Annotations and expression levels of transcripts involved in DNA conformation change in IMK and IMK + CAS relative to ASW.

Supplementary Table 3 Annotations and expression levels of transcripts involved in translation in IMK and IMK + CAS relative to ASW.

Supplementary Table 4 Annotations and expression levels of transcripts involved in cyclic nucleotide metabolic process in IMK and IMK + CAS relative to ASW.

Supplementary Table 5 Annotations and expression levels of transcripts involved in phosphorylation in IMK and IMK + CAS relative to ASW.

Supplementary Table 6 Annotations and expression levels of transcripts for ion transporters in IMK and IMK + CAS relative to ASW.

Supplementary Table 7 Annotations and expression levels of transcripts involved in microtubule-based movement in IMK and IMK + CAS relative to ASW.
