# Supplementary material for: Transcriptome Reprogramming of Symbiodiniaceae Breviolum minutum in Response to Casein Amino Acids Supplementation
Source: Front Physiol. 2020 Nov 19;11:574654. doi: 10.3389/fphys.2020.574654 (PMC7710908; doi:10.3389/fphys.2020.574654)
Supplement: Supplementary Figure 1 — SSB01 transcripts for GCN2, TOP2, and CENP-A under different culturing and symbiotic conditions. Transcript levels for General Control Nonderepressible 2 (GCN2, s6_33548), topoisomerase II (TOP2, s6_445) and histone h3-like centromeric protein a-like (CENP-A, s6_36342) expressed as transcripts per kilobase million(TPM) from in vitro cultures (ASW, IMK and CAS) and populating symbiotic SSB01 (12 d, 30 d, and steady state) inthe Aiptasia host. Results of symbiotic SSB01 were obtained by analyzing the data from Baumgarten et al., 2015; andXiang et al., 2020. Shown are means ± SDs from at least three biological replicates. [file Data_Sheet_1.pdf]

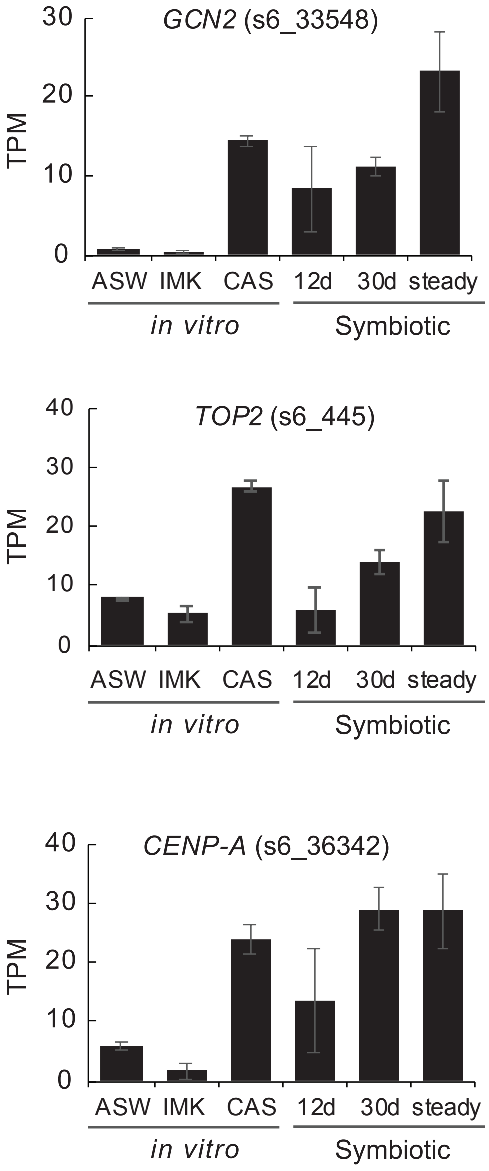

Supplementary Figure 1. SSB01 transcripts for GCN2, TOP2, and CENP-A under different culturing and symbiotic conditions. Transcript levels for General Control Nonderepressible 2 (GCN2, s6_33548), topoisomerase II (TOP2, s6_445) and histone h3-like centromeric protein a-like (CENP-A, s6_36342) expressed as transcripts per kilobase million (TPM) from *in vitro* cultures (ASW, IMK and CAS) and populating symbiotic SSB01 (12 d, 30 d, and steady state) in the Aiptasia host. Results of symbiotic SSB01 were obtained by analyzing the data from Baumgarten *et al*. 2015; and Xiang *et al*., 2020. Shown are means ± SDs from at least three biological replicates.

Supplemental Table 1. UniProt IDs of protein sequences used for amino acid-sequence alignment and phylogenetic tree.

ID	Description
s6_445	dna gyrase topoisomerase a subunit family protein (type II)
s6_4990	dna topoisomerase 2
s6_7490	dna topoisomerase 2
s6_800	dna topoisomerase 3-alpha
s6_2461	dna topoisomerase 3-beta-1
s6_190	dna topoisomerase III beta
s6_10839	dna topoisomerase family protein
s6_3028	dna topoisomerase II
s6_42823	dna topoisomerase I
s6_19109	topoisomerase III beta
s6_46657	mca1_debha (has a dna topoisomerase 2-like protein domain)
P30181	TOP1A_Arabidopsis
Q9FJ79	TOP1B_Arabidopsis
P30182	TOP2_Arabidopsis
Q9LVP1	TOP3A_Arabidopsis
F4ISQ7	TOP3B_Arabidopsis
Q9LZ03	TOP6A_Arabidopsis
P11387	TOP1_Human
P04786	TOP1_Yeast
P06786	TOP2_Yeast
P13099	TOP3_Yeast
P11388	TOP2A_Human
Q02880	TOP2B_Human
Q13472	TOP3A_Human
O95985	TOP3B_Human
Q5Q0E6	TO6BL_Arabidopsis
Q9CAF6	GYRA_Arabidopsis
Q9C5V6	TOP6B_Arabidopsis

Supplemental Table 2. Annotations and expression levels of transcripts involved in DNA conformation change in IMK and CAS relative to ASW.

ID	Annotation	Log2(Fold Change)	
		IMK	CAS
s6_36342	histone h3-like centromeric protein a-like	NS	1.840309839
s6_445*	dna gyrase topoisomerase a subunit family protein (type II)	NS	1.605050216
s6_4990*	dna topoisomerase 2	NS	1.475542553
s6_5055	condensin complex subunit 1	NS	1.437911577
s6_7490*	dna topoisomerase 2	NS	1.217750319
s6_800*	dna topoisomerase 3-alpha	NS	1.089148206
s6_2461*	dna topoisomerase 3-beta-1	NS	1.087360945
s6_190*	dna topoisomerase III beta	NS	1.08305918
s6_3357	structural maintenance of chromosomes 2 (SMC2)	NS	1.005647138
s6_10839*	dna topoisomerase family protein	NS	0.886451327
s6_5945	structural maintenance of chromosomes protein 1 (SMC1) like protein	NS	0.82016822
s6_2729	nucleosome assembly protein 1,1	NS	0.777967651
s6_3028*	dna topoisomerase II	NS	0.666457278
s6_42823*	dna topoisomerase I	NS	0.485587077
s6_19109*	topoisomerase III beta	NS	0.479841491
s6_46657	mca1_debha (has a dna topoisomerase 2-like protein domain)	NS	0.288942868
s6_4125	h3	NS	-0.390963094
s6_34360	histone h2a	NS	-0.661519768
s6_16949	histone h3	NS	-0.74061421
s6_638	structural maintenance of chromosomes 1 (SMC1)	NS	-0.86264299

NS = Not significant

Supplemental Table 3. Annotations and expression levels of transcripts involved in translation in IMK and CAS relative to ASW.

ID	Annotation	Log2(Fold Change)	
		IMK	CAS
s6_33548	eif2 alpha kinase gcn2	NS	4.242509
s6_1943	elongation factor tu	NS	1.84401
s6_1025	valine--trna ligase	NS	1.704732
s6_51857	eukaryotic translation initiation factor 3 subunit	NS	1.425556
s6_21694	seryl-trna synthetase	NS	1.218633
s6_6586	lysyl-trna synthetase	NS	1.193531
s6_877	eukaryotic translation initiation factor 4a	NS	1.117279
s6_15298	translation initiation factor if-2	NS	1.07941
s6_34150	hypothetical protein Pmar_PMAR005139	NS	1.003872
s6_9800	seryl-trna serine--trna	NS	0.947004
s6_3865	achain crystal structure of engineered northeast structural genomics consortium target	NS	0.878087
s6_40474	poly binding cytoplasmic isoform cra_c	NS	0.838509
s6_7607	alanyl-trna synthetase	NS	0.767272
s6_28691	elongation factor	NS	0.717606
s6_52019	hypothetical protein Pmar_PMAR028699	NS	0.640479
s6_6433	isoleucine trna	NS	0.618561
s6_3694	glutamate--trna ligase	NS	0.614944
s6_69	asparaginyl-trna synthetase	NS	0.613084
s6_10329	leucyl-trna cytoplasmic-like	NS	0.604945
s6_24243	prolyl-trna synthetase	NS	0.593424
s6_8934	chloroplast translation factor ts	NS	0.592851
s6_13292	histidyl-trna	NS	0.587008
s6_40676	50s ribosomal protein	NS	0.571693
s6_14811	ribosome recycling factor	NS	0.540693
s6_31340	rna helicase-1	NS	0.534643
s6_5496	ribosomal protein n-terminal domain containing protein	NS	0.530623
s6_51362	eukaryotic translation initiation	NS	0.507587
s6_245	lysyl-trna synthetase	NS	0.493046
s6_3048	glycyl-trna synthetase	NS	0.467294
s6_6170	riken cdna 2610011n19 gen	NS	0.460501
s6_34673	leucine--trna cytoplasmic	NS	0.445342
s6_12216	tryptophanyl-trna synthetase	NS	0.440592
s6_10417	elongation factor tu	NS	0.410769
s6_25344	aspartyl-trna synthetase	NS	0.38872
s6_23169	non-discriminatory gln-glu-trna synthetase	NS	0.370669
s6_33760	valyl trna synthetase	NS	0.359458

s6_33966	60s ribosomal protein	NS	-0.28204
s6_16477	glycyl-trna synthetase	NS	-0.31597
s6_984	eukaryotic translation initiation	NS	-0.33233
s6_34333	50s ribosomal protein l13	NS	-0.3402
s6_34952	lsu ribosomal protein l24p	NS	-0.34854
s6_5949	ribosomal protein mitochondrial	NS	-0.37972
s6_10622	phenylalanyl-trna synthetase beta chain	NS	-0.38861
s6_34927	eukaryotic peptide chain release	NS	-0.39972
s6_3187	ribosomal protein	NS	-0.40724
s6_32631	eukaryotic translation initiation	NS	-0.40935
s6_40633	glycyl-trna synthetase	NS	-0.41492
s6_9625	ribosomal protein l15	NS	-0.41835
s6_46745	60s ribosomal protein	NS	-0.41918
s6_53712	40s ribosomal protein	NS	-0.42157
s6_11490	Ribosomal protein L36	NS	-0.42526
s6_51269	30s ribosomal protein s13	NS	-0.42597
s6_32439	eukaryotic translation initiation factor 4e-like	NS	-0.43424
s6_51036	60s ribosomal protein	NS	-0.44817
s6_33483	60s ribosomal protein l29	NS	-0.458
s6_31435	60s ribosomal protein	NS	-0.46612
s6_31133	60s acidic ribosomal protein lp2	NS	-0.47092
s6_39736	50s ribosomal protein l14	NS	-0.47795
s6_15407	60s ribosomal protein l23a	NS	-0.49093
s6_26280	60s ribosomal protein	NS	-0.49229
s6_3805	seryl-trna	NS	-0.49294
s6_25684	translation initiation factor eif-1a	NS	-0.50022
s6_39167	ribosomal protein s21	NS	-0.50303
s6_28183	ribosomal protein s27a	NS	-0.5118
s6_40185	elongation factor	NS	-0.512
s6_24162	ribosomal protein l29	NS	-0.51635
s6_31416	50s ribosomal subunit l24	NS	-0.52363
s6_17156	hypothetical protein THAOC_31095	NS	-0.52536
s6_48138	60s ribosomal protein	NS	-0.52625
s6_36630	60s ribosomal protein	NS	-0.52861
s6_29281	ribosomal protein	NS	-0.53121
s6_36116	40s ribosomal protein	NS	-0.53771
s6_31751	40s ribosomal protein	NS	-0.54103
s6_38180	ribosomal protein	NS	-0.55223
s6_34592	ribosomal protein l18a	NS	-0.55451
s6_48933	40s ribosomal protein s16	NS	-0.55603
s6_1531	50s ribosomal protein l2	NS	-0.55794
s6_14372	50s ribosomal protein l11	NS	-0.56475
s6_40921	ribosomal protein l44	NS	-0.57265

s6_26347	ribosomal protein	NS	-0.57908
s6_27741	60s ribosomal protein	NS	-0.5791
s6_34411	translation initiation factor if-1	NS	-0.59041
s6_40242	eukaryotic translation initiation factor 3	NS	-0.59327
s6_21497	eukaryotic translation initiation factor 3 subunit	NS	-0.59631
s6_25721	mitochondrial ribosomal protein s8	NS	-0.59983
s6_34382	eukaryotic translation initiation	NS	-0.60095
s6_23858	50s ribosomal protein l17	NS	-0.6049
s6_35193	eukaryotic translation initiation factor 3 subunit k	NS	-0.61211
s6_41281	50s ribosomal protein l24	NS	-0.61664
s6_32906	60s ribosomal protein l43	NS	-0.62846
s6_183	eukaryotic translation initiation factor 4a	NS	-0.62998
s6_4671	ribosomal protein s27a	NS	-0.6369
s6_2973	40s ribosomal protein	NS	-0.64549
s6_30966	predicted protein	NS	-0.66081
s6_42717	60s ribosomal protein l39-like 5-like	NS	-0.6699
s6_26371	60s ribosomal protein l6	NS	-0.68172
s6_32071	60s ribosomal protein l38	NS	-0.686
s6_33393	60s ribosomal protein	NS	-0.69213
s6_31405	phenylalanyl-trna synthetase alpha	NS	-0.69798
s6_10921	40s ribosomal protein s19	NS	-0.71299
s6_32202	30s ribosomal protein s17	NS	-0.71709
s6_1945	40s ribosomal protein	NS	-0.71768
s6_37353	small subunit ribosomal protein 14	NS	-0.72653
s6_6001	30s ribosomal protein	NS	-0.72888
s6_39569	ribosomal protein s11	NS	-0.73265
s6_28386	40s ribosomal protein	NS	-0.73601
s6_25546	60s ribosomal protein	NS	-0.74147
s6_27737	40s ribosomal protein s8	NS	-0.75551
s6_29989	hypothetical protein Pmar_PMAR024716	NS	-0.7576
s6_39096	60s ribosomal protein l12	NS	-0.76454
s6_38166	ribosomal protein	NS	-0.77151
s6_26370	60S ribosomal protein L6E	NS	-0.77812
s6_3715	ubiquitin a-52 residue ribosomal protein fusion product 1	NS	-0.77938
s6_38901	60s ribosomal protein l13a	NS	-0.78213
s6_41050	ribosomal protein	NS	-0.78956
s6_40418	40s ribosomal protein s8	NS	-0.79182
s6_40914	40s ribosomal s14	NS	-0.79504
s6_32963	ribosomal protein component of cytosolic 80s ribosome and 60s large subunit	NS	-0.79689
s6_27641	50s ribosomal protein l23	NS	-0.79887
s6_39165	ribosomal protein l35	NS	-0.80028
s6_28154	60s ribosomal protein l22	NS	-0.80457

s6_53694	60s ribosomal subunit protein	NS	-0.80579
s6_38667	60s ribosomal protein	NS	-0.80789
s6_38673	40s ribosomal protein	NS	-0.81064
s6_27665	60s ribosomal protein l10a-	NS	-0.81074
s6_8479	60s ribosomal protein	NS	-0.81148
s6_5641	60s ribosomal protein	NS	-0.81369
s6_37881	60s ribosomal protein l38	NS	-0.81436
s6_4321	60s acidic ribosomal protein lp2	NS	-0.83937
s6_46766	ribosomal protein l22	NS	-0.84117
s6_2792	60s ribosomal protein	NS	-0.85442
s6_28121	40s ribosomal protein	NS	-0.8687
s6_12026	ribosomal protein l16	NS	-0.86983
s6_40774	ribosomal protein s27a	NS	-0.87122
s6_7050	60s ribosomal protein l33-	NS	-0.8734
s6_25911	ribosomal protein l22	NS	-0.87836
s6_29376	60s ribosomal protein l43	NS	-0.87856
s6_16140	ribosomal protein	NS	-0.88276
s6_16595	mitochondrial large subunit ribosomal protein	NS	-0.88852
s6_31770	60s ribosomal protein l10a-	NS	-0.89039
s6_3265	ribosomal protein l2	NS	-0.8972
s6_10600	ribosomal protein l18	NS	-0.90013
s6_39100	40s ribosomal protein	NS	-0.90019
s6_38452	40s ribosomal protein s27-	NS	-0.90134
s6_14822	60s ribosomal protein l26	NS	-0.90262
s6_48149	40s ribosomal protein x	NS	-0.90776
s6_31664	ribosomal protein l22	NS	-0.91792
s6_48126	ribosomal protein	NS	-0.92022
s6_38964	ribosomal protein l35	NS	-0.93954
s6_1599	60s ribosomal protein	NS	-0.94402
s6_28838	probable rpl43b-60s large subunit ribosomal protein	NS	-0.94944
s6_52510	60s ribosomal protein l34	NS	-0.94954
s6_11592	50s ribosomal protein l28	NS	-0.95415
s6_53684	ribosomal protein l21	NS	-0.9597
s6_758	40s ribosomal protein	NS	-0.961
s6_53742	60s ribosomal protein l12	NS	-0.97236
s6_51480	ribosomal protein component of cytosolic 80s ribosome and 60s	NS	-0.9729
s6_39751	60s ribosomal protein l12	NS	-0.97295
s6_22560	Ribosomal protein S14, conserved site	NS	-0.97986
s6_46793	ribosomal protein l13a	NS	-0.98261
s6_36110	60s ribosomal protein	NS	-0.98936
s6_36853	ribosomal protein	NS	-0.99281
s6_27740	40s ribosomal protein	NS	-0.99518
s6_38201	60s ribosomal protein l37	NS	-0.99656

s6_28989	40s ribosomal protein s0-	NS	-0.99811
s6_51344	60s ribosomal protein l34	NS	-1.00337
s6_50944	40s ribosomal protein	NS	-1.00549
s6_48125	ribosomal protein	NS	-1.009
s6_12874	40s ribosomal protein	NS	-1.01049
s6_30746	60s ribosomal protein l13a	NS	-1.01265
s6_37413	ribosomal protein s27a	NS	-1.0143
s6_35012	60s ribosomal protein l33-	NS	-1.01942
s6_28643	40s ribosomal protein	NS	-1.02194
s6_39087	ubiquitin-60s ribosomal protein l40-like isoform 2	NS	-1.02213
s6_4453	60s ribosomal protein l33-	NS	-1.0275
s6_36442	60s ribosomal protein	NS	-1.02831
s6_6061	60s ribosomal protein l37	NS	-1.02929
s6_34387	60s ribosomal protein	NS	-1.03058
s6_36998	60s ribosomal protein	NS	-1.032
s6_6232	40s ribosomal protein	NS	-1.03204
s6_26708	60s ribosomal protein	NS	-1.03355
s6_55679	60s ribosomal protein	NS	-1.03801
s6_55304	40s ribosomal protein	NS	-1.04057
s6_5609	ribosomal protein 29 40s small ribosomal subunit	NS	-1.05065
s6_23082	40s ribosomal protein	NS	-1.05377
s6_42704	40s ribosomal protein s15	NS	-1.05457
s6_24709	50s ribosomal protein	NS	-1.05562
s6_28010	40s ribosomal protein	NS	-1.05586
s6_9191	elongation factor	NS	-1.05905
s6_35857	60s ribosomal protein	NS	-1.05935
s6_52684	60s ribosomal protein l23a	NS	-1.06293
s6_25598	ribosomal protein s21	NS	-1.0647
s6_366	40s ribosomal protein s26-	NS	-1.06577
s6_35420	40s ribosomal protein	NS	-1.06984
s6_40704	40s ribosomal protein	NS	-1.07465
s6_53687	40s ribosomal protein	NS	-1.08281
s6_48169	ribosomal protein l18	NS	-1.08358
s6_46964	40s ribosomal protein	NS	-1.09781
s6_11408	ribosomal protein component of cytosolic 80s ribosome and 40s small subunit	NS	-1.10138
s6_51116	60s ribosomal protein	NS	-1.10504
s6_42399	40s ribosomal protein	NS	-1.11416
s6_39608	40s ribosomal protein s26-	NS	-1.11522
s6_23390	40s ribosomal protein	NS	-1.1235
s6_32094	60s ribosomal protein	NS	-1.12385
s6_51103	40s ribosomal protein	NS	-1.13124
s6_55206	40s ribosomal protein x	NS	-1.13508

s6_2390	60s ribosomal protein	NS	-1.13678
s6_31392	40s ribosomal protein s19	NS	-1.14349
s6_24298	ribosomal protein l21	NS	-1.15059
s6_2361	60s ribosomal protein l18a-	NS	-1.15273
s6_13942	40s ribosomal protein s15	NS	-1.16253
s6_53828	40s ribosomal protein s0-	NS	-1.16271
s6_30900	40s ribosomal protein s26-	NS	-1.17383
s6_36911	40s ribosomal protein s27-	NS	-1.19011
s6_31367	ribosomal protein component of cytosolic 80s ribosome and 40s	NS	-1.19074
s6_35077	40s ribosomal protein	NS	-1.19885
s6_51184	60s ribosomal protein	NS	-1.20238
s6_31753	60s ribosomal protein l37	NS	-1.20277
s6_40285	60s ribosomal protein	NS	-1.21391
s6_30773	40s ribosomal protein s0-	NS	-1.22109
s6_38264	60s ribosomal protein l10a-	NS	-1.22736
s6_53807	40s ribosomal protein	NS	-1.2286
s6_33719	ribosomal protein l18	NS	-1.22927
s6_27864	peptide chain release factor 1	NS	-1.23151
s6_31891	ubiquitin a-52 residue ribosomal protein fusion product 1	NS	-1.2405
s6_39224	component of cytosolic 80s ribosome and 60s large subunit	NS	-1.25943
s6_29130	60s ribosomal protein	NS	-1.28668
s6_39729	40s ribosomal protein	NS	-1.29733
s6_41657	ribosomal protein component of cytosolic 80s ribosome and 40s	NS	-1.32699
s6_703	60s ribosomal protein	NS	-1.3276
s6_5395	40s ribosomal protein s29-like	NS	-1.34213
s6_53743	60s ribosomal protein l34	NS	-1.35737
s6_15286	60s ribosomal protein	NS	-1.36654
s6_36617	60s ribosomal protein	NS	-1.38249
s6_39219	40s ribosomal protein s15	NS	-1.45956
s6_51072	ribosomal phosphoprotein	NS	-1.61353
s6_38195	translation initiation factor 5a	NS	-1.61924

NS = Not significant

Supplemental Table 4. Annotations and expression levels of transcripts involved in cyclic nucleotide metabolic process in IMK and CAS relative to ASW.

ID	Annotation	Log2(Fold Change)	
		IMK	CAS
s6_40118	adenylate guanylate cyclase with integral membran	NS	2.78401944
s6_22656	adenylate guanylate cyclase with gaf sensor and fh	NS	2.6666916
s6_40135	adenylate guanylate cyclase with integral membran	NS	1.92327721
s6_12083	Adenylyl cyclase class-3/4/guanylyl cyclase	NS	1.62641752
s6_7850	adenylate cyclase	NS	1.35867394
s6_9878	p25-alpha family protein	NS	1.33787639
s6_9596	adenylate cyclase 1	NS	1.32518058
s6_6330	adenylyl cyclase	NS	1.2656074
s6_4484	adenylate guanylate cyclase	NS	1.22227468
s6_28434	guanylate cyclase beta 1	NS	1.14067638
s6_7394	guanylate cyclase soluble subunit beta-2	NS	1.11808505
s6_15693	guanlylate cyclase	NS	0.94279261
s6_29427	signal transduction histidine kinase	NS	0.93207999
s6_8711	hypothetical protein	NS	0.89682325
s6_8273	adenylyl cyclase	NS	0.8583253
s6_40171	adenylyl cyclase	NS	0.82129304
s6_7007	adenylyl cyclase	NS	0.81293912
s6_7433	adenylate guanylate cyclase with integral membran	NS	0.76804944
s6_11617	chase2 domain protein	NS	0.75453081
s6_4274	guanylyl cyclase	NS	0.71035587
s6_2141	adenylyl cyclase	NS	0.5434649
s6_5101	adenylyl cyclase	NS	0.47922861
s6_10501	adenylate cyclase	NS	0.42236834
s6_27791	serine threonine rgc	NS	0.33239402
s6_9597	adenylate cyclase	NS	-0.7546788
s6_51552	adenylate guanylate cyclase with integral membran	NS	-1.4107271

NS = Not significant

Supplemental Table 5. Annotations and expression levels of transcripts involved in phosphorylation in IMK and CAS relative to ASW.

ID	Annotation	Log2(Fold Change)	
		IMK	CAS
s6_29014	3-phosphoinositide-dependent protein kinase-1	NS	-0.2970172
s6_9528	achain crystal structure of kinase domain of calciu	NS	0.45160336
s6_14659	agc family protein kinase	NS	-0.4529622
s6_42278	alpha kinase	NS	-1.7820837
s6_19353	alpha-glucan water dikinase	NS	1.29858263
s6_5312	alpha-glucan water dikinase	NS	0.87687741
s6_2517	alpha-glucan water dikinase 2	NS	1.2174411
s6_5935	ankyrin unc44	NS	1.56502548
s6_27927	atp synthase alpha	NS	0.43317488
s6_52041	atp synthase delta subunit	NS	0.52289262
s6_16600	atp synthase subunit b	NS	-0.4189052
s6_19485	aur protein kinase	NS	-0.7438986
s6_9157	aurora-like serine threonine protein kinase	NS	-0.6369279
s6_2516	br serine threonine-protein kinase	NS	0.68203421
s6_10751	ca2+ calmodulin-dependent protein kinase i-like p	NS	0.66483463
s6_13002	calcium calmodulin-dependent protein	NS	0.73119046
s6_17142	calcium calmodulin-dependent protein kinase	NS	1.9088002
s6_12210	calcium calmodulin-dependent protein kinase	NS	-0.8852656
s6_34043	calcium calmodulin-dependent protein kinase type	NS	2.06792567
s6_34534	calcium calmodulin-dependent protein kinase type	NS	1.03082566
s6_2960	calcium calmodulin-dependent protein kinase type	NS	0.7759898
s6_48302	calcium calmodulin-dependent protein kinase type	NS	0.68081322
s6_35513	calcium calmodulin-dependent protein kinase type	NS	-0.5228451
s6_14521	calcium calmodulin-dependent protein kinase type	NS	-0.576285
s6_4161	calcium calmodulin-dependent protein kinase type	NS	-0.560907
s6_33927	calcium dependent protein kinase	NS	3.34446722
s6_34663	calcium dependent protein kinase	NS	0.50018488
s6_23097	calcium dependent protein kinase 16	NS	-0.3308253
s6_29537	calcium-dependent protein	NS	3.02191675
s6_9931	calcium-dependent protein	NS	2.43027933
s6_13375	calcium-dependent protein	NS	2.41586879
s6_3854	calcium-dependent protein	NS	1.67555829
s6_2437	calcium-dependent protein	NS	1.64293709
s6_35045	calcium-dependent protein	NS	1.5552293
s6_1775	calcium-dependent protein	NS	1.5330225
s6_12850	calcium-dependent protein	NS	1.40396187

s6_33864	calcium-dependent protein	NS	1.3783706
s6_33623	calcium-dependent protein	NS	1.34367269
s6_3678	calcium-dependent protein	NS	1.3143613
s6_32754	calcium-dependent protein	NS	1.12885393
s6_6131	calcium-dependent protein	NS	1.11985275
s6_27792	calcium-dependent protein	NS	1.06504227
s6_32695	calcium-dependent protein	NS	1.06290068
s6_28331	calcium-dependent protein	NS	0.81124585
s6_3703	calcium-dependent protein	NS	0.80027077
s6_266	calcium-dependent protein	NS	0.7917278
s6_6874	calcium-dependent protein	NS	0.78527557
s6_13295	calcium-dependent protein	NS	0.77023468
s6_13355	calcium-dependent protein	NS	0.66690404
s6_29481	calcium-dependent protein	NS	0.66319956
s6_28973	calcium-dependent protein	NS	0.65282405
s6_34005	calcium-dependent protein	NS	0.64651064
s6_14731	calcium-dependent protein	NS	0.64089167
s6_16157	calcium-dependent protein	NS	0.61913442
s6_13624	calcium-dependent protein	NS	0.61273346
s6_1312	calcium-dependent protein	NS	0.58961873
s6_3000	calcium-dependent protein	NS	0.58904588
s6_31935	calcium-dependent protein	NS	0.53075361
s6_15599	calcium-dependent protein	NS	0.50973011
s6_797	calcium-dependent protein	NS	0.50242322
s6_7042	calcium-dependent protein	NS	0.4983566
s6_40503	calcium-dependent protein	NS	0.49151925
s6_604	calcium-dependent protein	NS	0.4775329
s6_13436	calcium-dependent protein	NS	0.42948172
s6_621	calcium-dependent protein	NS	0.40198055
s6_33726	calcium-dependent protein	NS	0.36409148
s6_1992	calcium-dependent protein	NS	-0.5116787
s6_1314	calcium-dependent protein	NS	-0.5156398
s6_6584	calcium-dependent protein	NS	-0.5535508
s6_24327	calcium-dependent protein	NS	-0.5771301
s6_8954	calcium-dependent protein	NS	-0.6120375
s6_32589	calcium-dependent protein kinase	NS	1.73812731
s6_51276	calcium-dependent protein kinase	NS	1.294519
s6_1525	calcium-dependent protein kinase	NS	0.89831989
s6_19358	calcium-dependent protein kinase	NS	-0.3926256
s6_30991	calcium-dependent protein kinase	NS	-0.53885
s6_40713	calcium-dependent protein kinase 7	NS	-0.6119753
s6_26556	calcium-dependent protein related	NS	-0.4830769
s6_5035	calmodulin-domain protein	NS	2.09873721

s6_32530	calmodulin-domain protein	NS	1.91265011
s6_23228	calmodulin-domain protein	NS	1.8908936
s6_33644	calmodulin-domain protein	NS	1.67055
s6_5407	calmodulin-domain protein	NS	1.59914707
s6_728	calmodulin-domain protein	NS	1.54835661
s6_1432	calmodulin-domain protein	NS	1.35699117
s6_33884	calmodulin-domain protein	NS	1.10372692
s6_10397	calmodulin-domain protein	NS	0.86537792
s6_8460	calmodulin-domain protein	NS	0.81958919
s6_23315	calmodulin-domain protein	NS	0.78058605
s6_23947	calmodulin-domain protein	NS	0.72044164
s6_30827	calmodulin-domain protein	NS	0.6334984
s6_52407	calmodulin-domain protein	NS	0.47365575
s6_34574	calmodulin-domain protein kinase	NS	0.81087301
s6_38119	calmodulin-domain protein kinase	NS	-0.6068509
s6_19742	calmodulin-domain protein kinase 1	NS	1.48976275
s6_8018	calmodulin-domain protein kinase 1	NS	1.1808073
s6_43243	camk family protein kinase	NS	1.46442331
s6_12254	camk family protein kinase	NS	1.02054439
s6_30001	camk family protein kinase	NS	0.56334968
s6_9261	camk family protein kinase	NS	0.55328423
s6_12845	camk family protein kinase	NS	-0.5392136
s6_3062	camp-dependent protein kinase catalytic	NS	0.72988595
s6_10042	camp-dependent protein kinase catalytic	NS	0.47354894
s6_35960	camp-dependent protein kinase catalytic	NS	-0.7666271
s6_24210	carbon catabolite derepressing protein	NS	-0.376029
s6_2554	carbon catabolite derepressing protein	NS	-0.3794785
s6_10937	carbon catabolite derepressing protein kinase	NS	0.92613408
s6_51845	casein kinase	NS	2.63479701
s6_11085	casein kinase	NS	2.34967385
s6_14170	casein kinase	NS	1.12632956
s6_33708	casein kinase	NS	1.08175353
s6_25157	casein kinase	NS	-0.4371571
s6_33926	casein kinase family protein	NS	1.02374004
s6_39233	casein kinase i isoform delta-like protein	NS	-0.5399455
s6_17154	cbl-interacting protein kinase	NS	-0.5061703
s6_2518	cbl-interacting serine threonine-protein kinase 19	NS	-0.5574288
s6_4289	ccaat-binding transcription factor subunit	NS	-0.8854114
s6_44909	cell division control protein 28	NS	0.70671593
s6_47074	cell division protein	NS	-1.2186022
s6_33594	cell division protein kinase	NS	-0.6707485
s6_2534	cgmp dependent protein kinase	NS	0.78797916
s6_28298	cgmp-dependent protein	NS	1.76559175

s6_2743	cgmp-dependent protein	NS	1.25254749
s6_13376	cgmp-dependent protein	NS	1.22211515
s6_16608	cgmp-dependent protein	NS	1.19781055
s6_50966	cgmp-dependent protein	NS	0.8489316
s6_10341	cgmp-dependent protein	NS	0.40312118
s6_35240	cgmp-dependent protein	NS	0.38180326
s6_2401	cgmp-dependent protein kinase	NS	1.80668286
s6_4214	cgmp-dependent protein kinase	NS	1.55438891
s6_35984	cgmp-dependent protein kinase	NS	0.82787612
s6_16253	cgmp-dependent protein kinase	NS	0.79935328
s6_35892	cgmp-dependent protein kinase 2-like	NS	-0.5931877
s6_22909	cgmp-dependent protein kinase egl-4-like isoform	NS	1.25684728
s6_3148	cgmp-dependent protein kinase i alpha	NS	0.50240891
s6_7176	cgmp-dependent protein kinase isozyme 1	NS	3.68407543
s6_28545	cgmp-dependent protein partial	NS	1.17443797
s6_584	cipk-like protein expressed	NS	1.52623437
s6_5219	cmgc cdk cdc2 protein kinase	NS	-0.7352868
s6_9524	cmgc family protein kinase	NS	1.043601
s6_5650	cmgc mapk family	NS	-0.4039184
s6_13098	cyclic gmp-dependent protein kinase	NS	0.8818908
s6_9206	Cyclic nucleotide-binding domain	NS	1.46343932
s6_28847	cyclin g-associated	NS	0.75542615
s6_25972	cyclin-dependent kinase 10	NS	-0.8616104
s6_6444	cyclin-dependent kinase 3	NS	1.85471703
s6_3707	cytidylate kinase	NS	0.98192521
s6_1529	diguanylate cyclase domain-containing protein	NS	0.41266129
s6_33671	dihydroxyacetone kinase	NS	0.68753127
s6_15094	domain and endonuclease exonuclease phospha[illegible]	NS	0.92554493
s6_33548	eif2 alpha kinase gcn2	NS	4.24250948
s6_5427	eukaryotic elongation factor-2 kinase	NS	-0.5677231
s6_22475	extracellular signal-regulated protein kinase	NS	-0.5381885
s6_10204	FAFL228Wp	NS	0.68101548
s6_29719	gliding motility related cam kinase	NS	0.9339328
s6_40066	ha-tagged protein kinase domain of mitogen-activ	NS	2.73432639
s6_23061	hunk protein	NS	0.65796322
s6_10927	hypothetical protein	NS	1.53331373
s6_16396	hypothetical protein	NS	0.68897905
s6_17224	hypothetical protein	NS	0.48520886
s6_2991	hypothetical protein	NS	0.48088429
s6_4704	hypothetical protein GUITHDRAFT_164559	NS	0.61852951
s6_4633	hypothetical protein GUITHDRAFT_40846, partia	NS	2.37937038
s6_18241	hypothetical protein GUITHDRAFT_40846, partia	NS	0.78484127
s6_16473	hypothetical protein GUITHDRAFT_76731	NS	1.03682049

s6_53918	hypothetical protein GUITHDRAFT_77982	NS	0.84888229
s6_12752	hypothetical protein NCAS_0C02830	NS	0.95114101
s6_7794	hypothetical protein THAOC_02564	NS	1.90279324
s6_18776	inositol monophosphatase	NS	-1.116603
s6_40592	Inositol polyphosphate-related phosphatase	NS	0.66212473
s6_833	integral membrane sensor hybrid histidine kinase	NS	0.87106904
s6_51174	kinase family protein	NS	-0.4319232
s6_4860	kinase-like protein	NS	1.67126355
s6_2902	kinase-like protein	NS	1.47615966
s6_4851	leucine-rich repeat and iq domain-containing prot	NS	1.21075459
s6_13864	map kinase-interacting serine threonine-protein	NS	0.78164284
s6_3407	map microtubule affinity-regulating kinase 2	NS	0.39323976
s6_8630	map microtubule affinity-regulating kinase 4	NS	0.32818059
s6_18527	member of the inositol monophosphatase protein	NS	0.85729017
s6_21784	mhck ef2 kinase domain family protein	NS	-2.293044
s6_28987	mitochondrial atp synthase f0 lipid binding subuni	NS	-0.6247175
s6_25052	mitochondrial pyruvate dehydrogenase kinase	NS	-0.3590392
s6_34465	mitogen activated protein kinase kinase kinase	NS	0.69179901
s6_14690	mitogen activated protein kinase kinase kinase 3	NS	0.46569548
s6_34371	mitogen-activated protein	NS	0.38953099
s6_18924	mitogen-activated protein kinase	NS	0.92320024
s6_9653	mitogen-activated protein kinase	NS	0.78859009
s6_7630	mitogen-activated protein kinase	NS	0.53364962
s6_29494	mitogen-activated protein kinase	NS	-0.4195409
s6_46758	mitogen-activated protein kinase	NS	-0.4897699
s6_8960	mitogen-activated protein kinase	NS	-0.7842464
s6_14851	mitogen-activated protein kinase 3	NS	1.16320372
s6_12915	myosin light chain kinase	NS	2.22167441
s6_25613	myosin light chain kinase	NS	1.4983274
s6_15791	myosin light chain kinase	NS	-0.754363
s6_54077	nek4 protein Serine/threonine-protein kinase Nek	NS	-1.1560179
s6_7303	nima-related kinase 7	NS	-1.1189461
s6_8707	non-specific serine threonine protein kinase	NS	1.54303663
s6_4011	nuak snf1-like 2	NS	0.96258434
s6_35474	nucleoside diphosphate kinase 7-like	NS	-0.4301505
s6_279	ovarian-specific serine threonine-protein kinase	NS	-0.3586034
s6_2888	Paramyosin, putative	NS	0.72445863
s6_51298	phosphate dikinase	NS	3.08808174
s6_33547	phosphate dikinase	NS	1.66346925
s6_14453	phosphatidylinositol 3- and 4-kinase domain-cont	NS	0.46559803
s6_27447	phosphatidylinositol 3- root isoform	NS	0.90313341
s6_34611	phosphoenolpyruvate carboxylase kinase	NS	1.5639676
s6_781	phosphoenolpyruvate synthase	NS	1.00762787

s6_6310	phosphoinositide 3-kinase regulatory subunit 4-lik	NS	1.54331577
s6_28626	phosphoinositide-dependent protein	NS	-0.4942233
s6_43078	phosphoinositide-dependent protein	NS	-0.7254442
s6_37659	pkinase-domain-containing protein	NS	0.90410173
s6_17873	probable serine threonine-protein kinase abkc-like	NS	0.515838
s6_20038	protein	NS	0.7016155
s6_3440	protein camp- catalytic chain	NS	0.4483526
s6_6542	protein cgmp- type i	NS	0.79150769
s6_15935	protein kinase	NS	2.81103367
s6_17584	protein kinase	NS	1.82337375
s6_5182	protein kinase	NS	1.65319195
s6_40107	protein kinase	NS	1.4560932
s6_40092	protein kinase	NS	1.11969923
s6_3686	protein kinase	NS	0.60772001
s6_9228	protein kinase	NS	0.52659247
s6_13785	protein kinase	NS	0.50438258
s6_293	protein kinase	NS	0.42988307
s6_5673	protein kinase	NS	0.38743948
s6_9204	protein kinase	NS	0.33289049
s6_3550	protein kinase	NS	-0.3953246
s6_1058	protein kinase	NS	-0.4248657
s6_9491	protein kinase	NS	-1.0535537
s6_4572	protein kinase 2	NS	0.69560651
s6_11842	protein kinase b-like protein	NS	0.4080949
s6_23199	protein kinase domain containing protein	NS	3.64839153
s6_6015	protein kinase domain containing protein	NS	2.17876764
s6_21915	protein kinase domain containing protein	NS	1.83006848
s6_243	protein kinase domain containing protein	NS	1.3502419
s6_12011	protein kinase domain containing protein	NS	1.17630465
s6_33753	protein kinase domain containing protein	NS	1.16139404
s6_34237	protein kinase domain containing protein	NS	1.07320252
s6_18025	protein kinase domain containing protein	NS	1.07137032
s6_43520	protein kinase domain containing protein	NS	0.88382998
s6_34765	protein kinase domain containing protein	NS	0.68426116
s6_12233	protein kinase domain containing protein	NS	0.64648863
s6_7045	protein kinase domain containing protein	NS	0.64067473
s6_8483	protein kinase domain containing protein	NS	0.54782934
s6_17670	protein kinase domain containing protein	NS	0.54632236
s6_35504	protein kinase domain containing protein	NS	0.48944526
s6_23886	protein kinase domain containing protein	NS	0.4822645
s6_13769	protein kinase domain containing protein	NS	0.47103171
s6_6007	protein kinase domain containing protein	NS	0.46533891
s6_40389	protein kinase domain containing protein	NS	0.45200921

s6_9651	protein kinase domain containing protein	NS	0.44066364
s6_38306	protein kinase domain containing protein	NS	-0.3780226
s6_29016	protein kinase domain containing protein	NS	-0.6278009
s6_27627	protein kinase domain containing protein	NS	-0.6574332
s6_24640	protein kinase domain containing protein	NS	-0.7005173
s6_14552	protein kinase domain containing protein	NS	-0.7993244
s6_13987	protein kinase domain containing protein	NS	-0.8577726
s6_8780	protein kinase domain protein	NS	0.6400958
s6_39440	protein kinase domain protein	NS	0.63185385
s6_880	protein kinase domain protein	NS	0.36681523
s6_40176	protein kinase domain-containing protein	NS	0.64667183
s6_23760	protein kinase domain-containing protein	NS	-0.6804664
s6_25010	protein kinase domain-containing protein	NS	-0.8580696
s6_15960	protein kinase family protein	NS	0.45926191
s6_20172	protein kinase family transcriptional partial	NS	2.93248973
s6_30180	protein kinase-like protein	NS	-0.7794892
s6_28864	protein-tyrosine kinase	NS	1.10043794
s6_3590	pyruvate dehydrogenase kinase	NS	-0.8543281
s6_732	pyruvate phosphate dikinase	NS	-0.5473402
s6_20965	rac-beta serine threonine-protein kinase-	NS	1.08604469
s6_1744	rac-beta serine threonine-protein kinase-	NS	-0.6142603
s6_4155	receptor-like protein kinase	NS	1.23343779
s6_9867	related to phosphatidylinositol 3-kinase	NS	0.99569876
s6_28568	ribosomal protein s6 kinase beta-1-like	NS	0.65395705
s6_2952	sensory box sensor histidine kinase	NS	0.94807466
s6_15834	serine threonine protein	NS	1.33452348
s6_1079	serine threonine protein	NS	0.67257359
s6_8650	serine threonine protein	NS	0.49323526
s6_10620	serine threonine protein	NS	0.36976562
s6_13954	serine threonine protein	NS	-0.9604627
s6_28044	serine threonine protein kinase	NS	1.85743107
s6_40741	serine threonine protein kinase	NS	0.87949099
s6_39354	serine threonine protein kinase	NS	0.65179762
s6_23253	serine threonine protein kinase	NS	0.44596038
s6_28231	serine threonine protein kinase	NS	0.39746252
s6_558	serine threonine protein kinase	NS	-0.3999104
s6_23700	serine threonine protein kinase	NS	-0.5000223
s6_15085	serine threonine protein kinase	NS	-0.6147977
s6_8490	serine threonine protein kinase	NS	-0.7991599
s6_25522	serine threonine protein kinase	NS	-0.949923
s6_14969	serine threonine protein kinase	NS	-0.9923991
s6_3844	serine threonine protein kinase ctr3	NS	1.61707072
s6_47667	serine threonine-protein kinase	NS	2.33279763

s6_1692	serine threonine-protein kinase	NS	1.52479218
s6_17485	serine threonine-protein kinase	NS	1.07587625
s6_4476	serine threonine-protein kinase	NS	0.53670748
s6_10789	serine threonine-protein kinase	NS	-0.4582742
s6_17667	serine threonine-protein kinase 16	NS	-0.4956416
s6_21594	serine threonine-protein kinase 6	NS	-0.7855981
s6_19468	serine threonine-protein kinase 9	NS	0.56829645
s6_1166	serine threonine-protein kinase chk1	NS	0.39639185
s6_10090	serine threonine-protein kinase dclk3-like	NS	-1.2339007
s6_42855	serine threonine-protein kinase h1	NS	1.8761985
s6_35897	serine threonine-protein kinase mark2- partial	NS	1.49779201
s6_34011	serine threonine-protein kinase nek4	NS	1.47869316
s6_13632	serine threonine-protein kinase nek5	NS	0.79845641
s6_29707	serine threonine-protein kinase nek8	NS	-0.5055104
s6_4614	serine threonine-protein kinase pepkr2	NS	2.0831067
s6_917	serine threonine-protein kinase pepkr2-like	NS	0.64987325
s6_9197	serine threonine-protein kinase prp4	NS	-0.5970636
s6_3261	serine_threonine protein kinase receptor	NS	1.16303221
s6_5296	serine-threonine protein	NS	0.4386825
s6_21540	serine-threonine protein	NS	-0.4620184
s6_30755	serine-threonine protein	NS	-0.6237931
s6_9002	serine-threonine protein plant-	NS	0.94709875
s6_16759	serine-threonine protein plant-	NS	0.35744191
s6_15725	serine-threonine protein plant-	NS	-0.5541655
s6_14887	serine-threonine protein plant-	NS	-0.6241852
s6_10567	serine-threonine protein plant-	NS	-0.6615726
s6_5284	Serine/threonine protein kinase	NS	0.42803452
s6_17793	shaggy-like kinase	NS	0.86809371
s6_29427	signal transduction histidine kinase	NS	0.93207999
s6_35980	sperm motility kinase w-like	NS	2.54745625
s6_13742	spindle assembly checkpoint kinase	NS	0.55393461
s6_40252	ste ste20 ysk protein kinase	NS	0.61472862
s6_15613	sterile alpha motif and leucine zipper containing k	NS	1.00418181
s6_3093	tccd-inducible-parp-like domain-containing proteir	NS	1.72539637
s6_38358	tkl family protein kinase	NS	0.45765026
s6_28141	tousled-like kinase 2	NS	0.3806002
s6_28627	two-component hybrid sensor and regulator	NS	0.81960126
s6_19424	two-component hybrid sensor and regulator	NS	0.65003391
s6_11018	unusual protein kinase	NS	0.97878047
s6_5393	unusual protein kinase	NS	0.68669431

NS = Not significant

Supplemental Table 6. Annotations and expression levels of transcripts for ion transporters in IMK and CAS relative to ASW.

ID	Annotation	Log2(Fold Change)	
		IMK	CAS
s6_6660	ion transport protein	NS	2.8464387
s6_29463	voltage-gated sodium channel	NS	2.82598409
s6_11186	chloride carrier channel family	NS	2.76361612
s6_34065	voltage-gated cation	NS	2.52669749
s6_35937	voltage-gated sodium channel protein	NS	2.41214047
s6_10391	cation channel family protein	NS	2.33943744
s6_5492	ammonium transporter	NS	2.27966248
s6_4371	potassium sodium hyperpolarization-activated cyclic nucl	NS	2.23081143
s6_32551	ammonium transporter	NS	2.03002431
s6_21960	ion transport protein	NS	2.01812916
s6_4379	sulfate transporter chloroplastic-like	NS	1.93734711
s6_40112	protein kinase domain containing protein	NS	1.93063866
s6_8054	Intracellular calcium-release channel	NS	1.84086236
s6_34137	voltage-gated sodium channel sns	NS	1.82978398
s6_2849	kef-type k+ transport membrane component	NS	1.82447968
s6_10871	voltage-gated sodium channel	NS	1.80589404
s6_17047	p-type atpase superfamily	NS	1.80416035
s6_39252	bacteriorhodopsin ii	NS	1.80332134
s6_7627	sulfate bicarbonate oxalate exchanger and transporter sa	NS	1.79817624
s6_27994	gamma-aminobutyric acid receptor subunit gamma-3	NS	1.74858177
s6_5924	p-type atpase superfamily	NS	1.73452494
s6_21873	cyclic nucleotide-binding protein	NS	1.72686004
s6_35189	sodium channel protein type 11 subunit alpha-like	NS	1.70457384
s6_12125	isoform cra_a	NS	1.70414662
s6_16819	voltage-gated ion channel superfamily	NS	1.70253606
s6_34545	ion transport protein	NS	1.67860229
s6_5094	conserved hypothetical protein	NS	1.64006639
s6_7107	conserved hypothetical protein	NS	1.63649597
s6_36139	conserved unknown protein	NS	1.63407133
s6_12331	voltage-gated sodium channel	NS	1.61693804
s6_54172	sodium channel protein type 5 subunit alpha	NS	1.59783032
s6_33766	cre-eat-6 protein	NS	1.58074418
s6_12982	potassium sodium hyperpolarization-activated cyclic nucl	NS	1.57844297
s6_5792	glycoside pentoside hexuronide transporter	NS	1.5749291
s6_16850	voltage-gated sodium channel	NS	1.55535324
s6_17045	zip family transporter	NS	1.55129124
s6_12085	cation channel family protein	NS	1.54044047

s6_40058	voltage-gated sodium channel	NS	1.49763215
s6_8585	sodium calcium	NS	1.47013781
s6_28921	akt2 inward rectifier channel	NS	1.46274641
s6_3745	membrane transport	NS	1.45546719
s6_20127	af484082_1voltage-dependent non-l-type calcium channe	NS	1.45395036
s6_9766	sodium calcium transport	NS	1.44575418
s6_9016	sodium calcium transport	NS	1.44510915
s6_1219	sodium channel protein	NS	1.43977779
s6_1055	voltage-gated ion channel superfamily	NS	1.42606522
s6_29162	voltage-gated sodium channel	NS	1.42376054
s6_5892	voltage-gated cation	NS	1.41421656
s6_21563	hyperpolarizaion-activated cyclic nucleotide-gated cation	NS	1.38519496
s6_25858	sodium channel protein type 11 subunit alpha	NS	1.36867165
s6_15330	voltage-gated cation	NS	1.32337803
s6_34567	cre-nnt-1 protein	NS	1.32218611
s6_18280	conserved hypothetical protein	NS	1.31104541
s6_11807	na+ h+ antiporter	NS	1.30734444
s6_11073	ion transport protein	NS	1.30416571
s6_28906	phospholipid-transporting atpase 3	NS	1.30060699
s6_6762	cation transporter component	NS	1.29053702
s6_33321	nad transhydrogenase-like	NS	1.28442805
s6_11102	vacuolar proton translocating atpase 116 kda subunit a	NS	1.27134422
s6_9827	voltage-gated ion channel superfamily	NS	1.26526781
s6_5869	voltage-gated ion channel superfamily	NS	1.25589084
s6_6765	conserved hypothetical protein	NS	1.2459151
s6_18284	conserved hypothetical protein	NS	1.23082173
s6_28642	voltage-gated ion channel superfamily	NS	1.22989321
s6_40453	voltage-gated ion channel superfamily	NS	1.22763986
s6_9489	conserved hypothetical protein	NS	1.22676413
s6_9432	ascidian calcium channel alpha1-subunit	NS	1.22516049
s6_40258	sodium channel protein type 2 subunit alpha-like	NS	1.22075371
s6_3334	guanylate cyclase activator 1a	NS	1.21884566
s6_23860	chloride channel	NS	1.21012604
s6_26520	conserved hypothetical protein	NS	1.20022662
s6_12787	voltage-gated ion channel superfamily	NS	1.19963286
s6_18506	hyperpolarization-activated cyclic nucleotide-modulated c	NS	1.1776613
s6_9337	sulfate transporter	NS	1.16901244
s6_6593	p-type atpase superfamily	NS	1.16043836
s6_10943	potassium channel	NS	1.15318553
s6_8391	sodium calcium	NS	1.14640118
s6_33576	sodium hydrogen exchanger	NS	1.14555376
s6_14742	voltage-gated ion channel superfamily	NS	1.14400084
s6_2885	two pore calcium channel protein 1-like	NS	1.13910797

s6_34408	conserved hypothetical protein	NS	1.13519553
s6_24018	ion transport protein	NS	1.13498192
s6_28123	voltage-gated ion channel superfamily	NS	1.1320583
s6_18800	transient receptor potential cation channel subfamily m m	NS	1.13074346
s6_692	neuronal acetylcholine receptor subunit alpha-2	NS	1.12995787
s6_2406	p-type partial	NS	1.12898958
s6_33540	hypothetical protein Pmar_PMAR028239	NS	1.12804186
s6_34647	bsc1 sodium channel protein	NS	1.1271673
s6_11034	ion transport protein	NS	1.12595912
s6_8007	conserved hypothetical protein	NS	1.11937307
s6_7232	voltage-dependent l-type calcium channel subunit alpha-[illegible]	NS	1.11313214
s6_34227	voltage-dependent n-type calcium channel	NS	1.10974178
s6_8690	hypothetical protein Pmar_PMAR007653	NS	1.10730094
s6_36059	ion transport protein	NS	1.10444211
s6_11040	ion transport protein	NS	1.10389289
s6_33819	p-type	NS	1.10007797
s6_1731	voltage-gated sodium channel	NS	1.09423646
s6_42934	conserved hypothetical protein	NS	1.09306727
s6_9836	p-type atpase	NS	1.08747508
s6_34701	cation channel family protein	NS	1.08396755
s6_4962	ion transport protein	NS	1.0804369
s6_12456	sodium voltage- type alpha-like	NS	1.07973548
s6_31475	ion transport protein	NS	1.07722817
s6_724	conserved hypothetical protein	NS	1.07598841
s6_3890	cl- channel voltage-gated family protein	NS	1.07541605
s6_28350	cgmp-gated cation channel	NS	1.07449257
s6_7278	ion transport protein	NS	1.07201022
s6_9034	cl- channel voltage-gated family protein	NS	1.06578802
s6_658	voltage-dependent cation channel sc1	NS	1.0477021
s6_4562	voltage-gated cation	NS	1.03744828
s6_14828	cation channel family protein	NS	1.03721915
s6_6594	kef-type k+ transport system protein	NS	1.03022773
s6_17363	conserved hypothetical protein	NS	1.02917037
s6_16675	na+ h+ antiporter	NS	1.02613598
s6_10604	ion transport protein	NS	1.01584977
s6_10482	voltage-gated ion channel superfamily	NS	1.01387886
s6_7299	h(+) cl(-) exchange transporter 3-like	NS	1.0126442
s6_28065	k+ kef-type	NS	1.0086323
s6_12582	guanylate cyclase activator protein 1	NS	1.00730331
s6_356	ion transporter	NS	1.00489589
s6_39175	voltage-gated ion channel superfamily	NS	1.00478002
s6_7895	h+ transporting atpase	NS	0.98818532
s6_25472	cation channel family protein	NS	0.98304141

s6_17079	monovalent cation:proton antiporter-2 family	NS	0.97088743
s6_17718	potassium sodium hyperpolarization-activated cyclic nucl	NS	0.96532904
s6_27563	two-pore calcium channel	NS	0.9549112
s6_2252	ion transport protein	NS	0.9456101
s6_7125	potassium voltage-gated channel subfamily c member 2	NS	0.94518484
s6_7037	voltagegated ion channel superfamily	NS	0.94069392
s6_2007	bach_halss ame: full=halorhodopsin short=hr	NS	0.93734283
s6_8648	voltage-gated sodium channel	NS	0.9332013
s6_40409	sodium calcium	NS	0.93152843
s6_2111	potassium sodium hyperpolarization-activated cyclic nucl	NS	0.93026789
s6_9745	cgmp-gated cation channel	-2.7437142	0.92832765
s6_4152	cyclic nucleotide-binding protein	NS	0.92433236
s6_1906	sodium channel protein type 3 subunit alpha	NS	0.92044672
s6_29438	cgmp-gated cation channel	NS	0.90908166
s6_6880	cation transporter	NS	0.90541203
s6_33495	conserved hypothetical protein	NS	0.90348603
s6_967	anion exchanger family	NS	0.90031267
s6_14502	cation channel family protein	NS	0.8986878
s6_154	bach_halsd ame: full=halorhodopsin short=hr	NS	0.89377339
s6_43201	cation transporter component	NS	0.89266963
s6_2117	v-type h(+)-translocating pyrophosphatase	NS	0.89212066
s6_10067	sodium channel na bp	NS	0.88778666
s6_15528	cation channel family protein	NS	0.88528095
s6_6134	potassium partial	NS	0.87644667
s6_4786	cyclic nucleotide-binding protein	NS	0.87509484
s6_29319	ion transport protein	NS	0.87389391
s6_574	ae family transporter: anion exchange	NS	0.87383234
s6_31238	voltage-gated sodium channel	NS	0.87359576
s6_833	integral membrane sensor hybrid histidine kinase	NS	0.87106904
s6_9104	ion transport protein	NS	0.8661028
s6_33900	monovalent cation:proton antiporter-2 family	NS	0.86206644
s6_8273	adenylyl cyclase	NS	0.8583253
s6_34784	calcium voltage- t alpha 1i partial	NS	0.85743856
s6_21331	voltage-gated clc-type chloride	NS	0.85710605
s6_11426	ca- isoform d	NS	0.85498472
s6_28285	potassium voltage-gated channel subfamily a member 1	NS	0.83509212
s6_553	hypothetical protein	NS	0.82851378
s6_34278	cation channel family protein	NS	0.8268937
s6_40171	adenylyl cyclase	NS	0.82129304
s6_28627	two-component hybrid sensor and regulator	NS	0.81960126
s6_7007	adenylyl cyclase	NS	0.81293912
s6_27413	atp synthase alpha	NS	0.81113824
s6_9861	zinc transporter zupt	NS	0.80927208

s6_1540	nad mitochondrial precursor	NS	0.79960935
s6_7783	ammonium transporter	NS	0.79717709
s6_13413	two-pore calcium channel	NS	0.78814868
s6_688	monovalent cation:proton antiporter1 family	NS	0.78688109
s6_18775	h(+) cl(-) exchange transporter 7-like	NS	0.77895844
s6_35461	voltage-dependent sodium channel	NS	0.77131921
s6_7433	adenylate guanylate cyclase with integral membrane sens	NS	0.76804944
s6_6468	potassium voltage-gated channel subfamily h member 2	NS	0.7673147
s6_8545	voltage-gated sodium channel	NS	0.76584311
s6_7809	trp cation partial	NS	0.76438341
s6_8342	cyclic nucleotide-binding protein	NS	0.75892966
s6_6083	plasma-membrane proton-efflux p-type atpase	NS	0.75351257
s6_23514	conserved hypothetical protein	NS	0.75179057
s6_34132	voltage-gated sodium channel	NS	0.75012308
s6_29805	potassium channel protein	NS	0.7494864
s6_12784	potassium channel	NS	0.73843481
s6_12734	rhodopsin 3 partial	NS	0.73718346
s6_5457	voltage-dependent t-type calcium channel subunit alpha-	NS	0.72822613
s6_4472	voltage-gated ion channel superfamily	NS	0.7146868
s6_18876	ion transport protein	NS	0.71237052
s6_40330	voltage-gated ion channel superfamily	NS	0.70662043
s6_8601	voltage-gated sodium channel	NS	0.70395803
s6_11468	conserved hypothetical protein	NS	0.68934186
s6_4110	conserved hypothetical protein	NS	0.68710278
s6_8469	conserved unknown protein	NS	0.68426877
s6_9579	cation channel family protein	NS	0.68424283
s6_17344	conserved hypothetical protein	NS	0.68384285
s6_8933	potassium sodium hyperpolarization-activated cyclic nucl	NS	0.68009068
s6_8705	voltage-gated ion channel superfamily	NS	0.67505442
s6_34404	kef-type k+ transport nad-binding component	NS	0.67138378
s6_16502	mitochondrial phosphate carrier protein	NS	0.66988424
s6_6881	p-type transporter	NS	0.66956895
s6_12748	natural resistance-associated macrophage protein	NS	0.66556366
s6_28024	potassium sodium hyperpolarization-activated cyclic nucl	NS	0.66479231
s6_15718	ion transporter	NS	0.65971476
s6_19424	two-component hybrid sensor and regulator	NS	0.65003391
s6_9948	sulfate transporter family protein	NS	0.64655109
s6_40344	cation channel family protein	NS	0.64438148
s6_32575	cation transporter	NS	0.63098465
s6_4582	sodium calcium	NS	0.62401248
s6_51410	p-type atpase superfamily	NS	0.61852412
s6_593	potassium voltage-gated channel subfamily b member 2	NS	0.61174514
s6_12176	bacterial type voltage activated sodium channel	NS	0.60780227

s6_6294	voltage-gated ion channel superfamily	NS	0.59516994
s6_10776	voltage-gated sodium channel	NS	0.59515289
s6_34768	cl- channel voltage-gated family protein	NS	0.58873721
s6_34586	calcium-binding protein cml19	NS	0.58419813
s6_23184	hypothetical protein Pmar_PMAR007653	NS	0.58392591
s6_15135	k channel inward rectifier conserved region 2 domain prot	NS	0.58092401
s6_9220	v-type h(+)-translocating pyrophosphatase	NS	0.5784801
s6_3166	potassium channel	NS	0.57511166
s6_51101	cation channel family	NS	0.57190297
s6_10826	sodium voltage- type isoform cra_a	NS	0.56508563
s6_51680	vacuolar proton translocating atpase 116 kda subunit a	NS	0.56297969
s6_10859	Sodium/potassium-transporting ATPase subunit alpha	NS	0.56131727
s6_27953	hypothetical protein	NS	0.56103653
s6_23645	polycystin cation channel family	NS	0.55021777
s6_17358	chloride carrier channel family	NS	0.54926111
s6_35781	voltage-dependent cation channel	NS	0.54636942
s6_2141	adenylyl cyclase	NS	0.5434649
s6_3510	voltage-gated sodium channel subunit	NS	0.52743623
s6_2962	calcium atpase serca-like	NS	0.52430676
s6_52041	atp synthase delta subunit	NS	0.52289262
s6_34242	voltage-gated sodium channel subunit	NS	0.5201597
s6_14363	cation channel family	NS	0.51197569
s6_30734	voltage-gated sodium channel	NS	0.50728233
s6_40493	bacterial type voltage activated sodium channel	NS	0.50720749
s6_4728	probable phospholipid-transporting atpase ib	NS	0.49424414
s6_23079	hypothetical protein Pmar_PMAR007873	NS	0.49393961
s6_6589	voltage-gated sodium channel	NS	0.49387162
s6_29054	sugar (glycoside-pentoside-hexuronide) transporter	NS	0.49160526
s6_12170	ion transporter	NS	0.48437988
s6_9439	voltage-gated sodium channel	NS	0.48099045
s6_15285	sulfate transporter chloroplastic-like	NS	0.47825485
s6_6162	ion transport protein	NS	0.47807381
s6_25886	sodium transporter	NS	0.47313469
s6_40461	cation channel family	NS	0.47174295
s6_29638	protein kvs- isoform a	NS	0.46431479
s6_15519	cation channel family protein	NS	0.45776298
s6_15158	ion transport protein	NS	0.45716664
s6_2833	polycystic kidney disease 2-like 1 protein	NS	0.45045834
s6_4666	calcium-activated potassium channel alpha	NS	0.44909732
s6_29731	conserved hypothetical protein	NS	0.4482654
s6_932	h(+) cl(-) exchange transporter 5 isoform 3	NS	0.44366115
s6_12195	ion transport protein	NS	0.44271306
s6_10900	ion transporter	NS	0.44226007

s6_14362	cation channel family protein	NS	0.4405704
s6_3499	v-type h(+)-translocating pyrophosphatase	NS	0.43836037
s6_13798	kiaa1120 protein	NS	0.43542445
s6_27927	atp synthase alpha	NS	0.43317488
s6_17762	conserved hypothetical protein	NS	0.4329047
s6_41129	voltage-gated shaker-like k+ channel kcna	NS	0.42377544
s6_40155	voltage-gated sodium channel	NS	0.4215363
s6_15785	copper transporter atpase	NS	0.42063128
s6_6770	calcium atpase serca-like	NS	0.42022928
s6_5379	ion transport protein	NS	0.41654267
s6_14573	cation channel family protein	NS	0.40495881
s6_7072	metal ion transporter family	NS	0.38446622
s6_35963	sodium potassium calcium exchanger 2	NS	0.3738891
s6_3774	na+ h+ antiporter	NS	0.3728177
s6_962	bach_hals4 ame: full=halorhodopsin short=hr	NS	0.3662139
s6_7168	hypothetical protein Pmar_PMAR007653	NS	0.36602591
s6_10422	mg2+ transporter	NS	0.36589018
s6_3621	cyclic nucleotide-gated cation channel cnga1-3 and relate	NS	0.36569243
s6_13751	conserved hypothetical protein	NS	0.36411314
s6_11069	metal ion transporter family	NS	0.35904354
s6_35280	k+ kef-type	NS	0.35587754
s6_10923	conserved hypothetical protein	NS	0.35581723
s6_33522	ion transport protein	NS	0.35299476
s6_16845	voltage-gated ion channel superfamily	NS	0.34336497
s6_28581	hypothetical protein Pmar_PMAR007873	NS	0.33857157
s6_2246	vacuolar atp synthase subunit	NS	0.31021911
s6_8097	vacuolar atp synthase 21 kda proteolipid	NS	-0.3077948
s6_35890	anion exchanger family	NS	-0.308735
s6_2861	major facilitator superfamily protein	NS	-0.3143427
s6_770	atp synthase cf1 epsilon subunit	NS	-0.3242933
s6_35663	cation diffusion facilitator family transporter containing prc	NS	-0.3259512
s6_20915	atp synthase cf0 b chain subunit ii	NS	-0.329613
s6_30706	ammonium transporter	NS	-0.3319845
s6_38002	chloroplast atp synthase subunit c	NS	-0.3328191
s6_2894	copper-transporting atpase p-	NS	-0.3330715
s6_28419	ion transport protein	NS	-0.351526
s6_11045	ion transport protein	NS	-0.3528412
s6_39084	atp synthase cf1 delta subunit	NS	-0.3557616
s6_10705	PREDICTED: uncharacterized protein LOC762549 isofor	NS	-0.3669405
s6_529	atp synthase gamma	NS	-0.378749
s6_20522	kef-type k+ transport nad-binding component	NS	-0.380786
s6_25002	mitochondrial atp synthase f0 lipid binding subunit-like prc	NS	-0.3844164
s6_15803	voltage-gated sodium channel	NS	-0.3852357

s6_38325	conserved hypothetical protein	NS	-0.3875655
s6_15103	ion transport protein	NS	-0.3878355
s6_5045	ammonium transporter	NS	-0.3999749
s6_28365	chromate ion transporter family	NS	-0.4033255
s6_27594	bile acid:sodium symporter family protein	NS	-0.4103607
s6_18455	metal tolerance protein	NS	-0.4108835
s6_38413	calcium-dependent protein kinase 1	NS	-0.4188616
s6_16600	atp synthase subunit b	NS	-0.4189052
s6_34156	voltage-dependent l-type calcium channel subunit alpha-'	NS	-0.4207096
s6_691	coiled-coil domain containing 2-like	NS	-0.4408709
s6_6610	cation channel family protein	NS	-0.4484339
s6_40230	mitochondrial atp synthase f0 lipid binding subunit-like pro	NS	-0.450126
s6_2533	conserved hypothetical protein	NS	-0.454043
s6_23580	voltage-gated sodium channel	NS	-0.4542158
s6_31174	ion transport protein	NS	-0.4546497
s6_51708	vacuolar atp synthase subunit	NS	-0.4570501
s6_14724	cation channel family	NS	-0.4607199
s6_35136	na+ h+ antiporter	NS	-0.462022
s6_9039	mitochondrial atp synthase f1 delta subunit	NS	-0.4651426
s6_30713	zip family transporter	NS	-0.4671593
s6_23295	conserved hypothetical protein	NS	-0.4719866
s6_7410	potassium channel homolog	NS	-0.477947
s6_5173	mitochondrial import receptor subunit tom40	NS	-0.4824486
s6_38354	atp synthase alpha sodium ion specific	NS	-0.4907408
s6_29765	calcium ion transporter	NS	-0.4925507
s6_38776	atp synthase subunit	NS	-0.493059
s6_23784	ctr2 family transporter: copper ion ctr-type copper transpc	NS	-0.4935988
s6_496	voltage-gated sodium channel subunit	NS	-0.4941121
s6_7654	kef-type k+ transport nad-binding component	NS	-0.5148919
s6_14197	organic cation transporter	NS	-0.5158891
s6_31470	mg co ni transporter	NS	-0.5175903
s6_3042	voltage-gated sodium channel	-1.9112445	-0.5176598
s6_16257	zip-family zinc transporter	NS	-0.529447
s6_18919	hydrogen-transporting atp synthase	NS	-0.5353968
s6_52007	magnesium transporter nipa2-like	NS	-0.5391362
s6_42884	ion transporter	NS	-0.5429783
s6_5828	calcium proton exchanger family protein	NS	-0.5577977
s6_16538	ca2+ h+ antiporter	NS	-0.56349
s6_39280	atp synthase cf0 subunit i	NS	-0.5801545
s6_39443	atp f1 epsilon subunit	NS	-0.5838998
s6_9683	cac1m_musdo ame: full=muscle calcium channel subunit	NS	-0.5844276
s6_28099	k+-dependent na+ ca+ exchanger-like protein	NS	-0.5846465
s6_28286	voltage dependent anion channel	NS	-0.5863411

s6_3780	hypothetical protein Pmar_PMAR026893	NS	-0.5863805
s6_34739	divalent cation transporter	NS	-0.5876841
s6_14404	ammonium transporter	NS	-0.5902464
s6_28128	sss family transporter: sodium ion pantothenate	NS	-0.5929553
s6_10508	conserved hypothetical protein	NS	-0.5937731
s6_18229	potassium voltage-gated channel subfamily b member 1-l	NS	-0.6050435
s6_39193	opsin 2	NS	-0.6179683
s6_28987	mitochondrial atp synthase f0 lipid binding subunit-like pro	NS	-0.6247175
s6_14941	anion transporter	NS	-0.6303462
s6_24860	conserved hypothetical protein	NS	-0.6387953
s6_2570	organic cation transporter	NS	-0.6403529
s6_22045	ankyrin repeat-containing	NS	-0.6502889
s6_21501	inwardly rectifying k+	NS	-0.6595286
s6_16818	vacuolar atp synthase 16 kda proteolipid	NS	-0.6629688
s6_11696	cobalt and zinc h(+)-k(+) antiporter	NS	-0.6637049
s6_7609	atp-binding cassette sub-family b member mitochondrial	NS	-0.6663216
s6_5355	sco1 domain-containing protein	NS	-0.671252
s6_10828	solute carrier family 39 protein	NS	-0.6831401
s6_5720	sulfate permease family	NS	-0.686359
s6_16527	bacr3_halsd ame: full=archaerhodopsin-3 short=ar 3 flag	NS	-0.6960523
s6_6943	inorganic phosphate	NS	-0.714871
s6_241	voltage-dependent l type calcium channel alpha 1	NS	-0.7151408
s6_39162	atp synthase cf0 a chain	NS	-0.7228941
s6_33764	ammonium transporter	NS	-0.7358
s6_12483	neurotransmitter-gated ion-channel ligand-binding protein	NS	-0.7416037
s6_12614	bacr3_halsd ame: full=archaerhodopsin-3 short=ar 3 flag	NS	-0.7549905
s6_14575	hypothetical protein Pmar_PMAR007873	NS	-0.7888792
s6_17662	bile acid:na+ symporter family	NS	-0.7890782
s6_57443	ammonium	NS	-0.8661234
s6_11015	potassium channel tetramerisation domain containing	NS	-0.8668746
s6_12163	major facilitator superfamily protein	NS	-0.8703501
s6_21330	sulfate transporter	NS	-0.8774009
s6_16167	divalent cation transporter	NS	-0.8793441
s6_38230	chloroplast atp synthase	NS	-0.8824994
s6_11747	mg2+ transporter-e family	NS	-0.88779
s6_16652	af493793_1 sulphate transporter	NS	-0.8896658
s6_14671	ion transport protein	NS	-0.8911079
s6_50930	rhodopsin	NS	-0.9244223
s6_712	vacuolar atp synthase 16 kda proteolipid	NS	-0.9326315
s6_6985	ammonium	NS	-0.9352517
s6_48896	vacuolar atp synthase 16 kda proteolipid	NS	-0.9425393
s6_41226	nitrate transporter	NS	-0.9546087
s6_422	nitrate transporter	NS	-0.9640377

s6_13254	membrane protein	NS	-0.9780839
s6_43072	solute carrier family 22 member 15-like	NS	-1.0047216
s6_30780	major intrinsic protein	NS	-1.0370435
s6_51634	solute carrier family 22 member 8	NS	-1.0518534
s6_56281	ammonium transporter	NS	-1.1048015
s6_38207	ammonium transporter	NS	-1.112236
s6_34430	ammonium transporter	NS	-1.1564765
s6_47531	ankyrin unc44	NS	-1.1578197
s6_43218	major facilitator superfamily mfs_1	NS	-1.2311367
s6_40239	ammonium transporter	NS	-1.2545666
s6_420	nitrate transporter	NS	-1.4734192
s6_58513	nitrate transporter	NS	-1.5835972
s6_50951	ammonium transporter	NS	-1.708212
s6_44035	ammonium transporter	NS	-1.8317058
s6_55422	ammonium transporter	NS	-1.928497
s6_51281	ammonium transporter	NS	-1.9904485
s6_51578	ammonium transporter	-2.0523849	-2.2197376
s6_53768	ammonium transporter channel family	NS	-2.2449884
s6_51076	ammonium transporter partial	NS	-2.4735334
s6_38239	ammonium transporter channel family	NS	-3.1721517

NS = Not significant

Supplemental Table 7. Annotations and expression levels of transcripts involved in microtubule-based movement in IMK and CAS relative to ASW.

ID	Annotation	Log2(Fold Change)	
		IMK	CAS
s6_51770	chromosome-associated kinesin	NS	2.63045619
s6_15835	kcbp-like kinesin	NS	2.12174031
s6_40067	achain structure of the complex of a mitotic kinesin with its calcium binding regulator	NS	2.10333916
s6_27873	kinesin-like protein	NS	1.75159453
s6_9802	kinesin motor domain containing protein	NS	1.24912324
s6_28464	kinesin-like protein	NS	1.24640948
s6_11846	kinesin-like protein	NS	1.2025403
s6_28322	kinesin heavy chain	NS	1.17546377
s6_39168	kinesin motor domain containing protein	NS	1.17524743
s6_528	kcbp-like kinesin	NS	1.09900713
s6_635	kinesin heavy	NS	1.05016815
s6_2265	kinesin-like protein	NS	1.03257402
s6_31375	kinesin heavy chain	NS	1.00690238
s6_10057	kinesin-related protein 1	NS	0.98496459
s6_40269	kinesin	NS	0.98001726
s6_3349	kif21a protein	NS	0.97114248
s6_9976	kif13b protein	NS	0.93326282
s6_5879	kinesin heavy	NS	0.87271831
s6_9753	kinesin heavy	NS	0.86497447
s6_5213	kinesin motor domain containing protein	NS	0.86174511
s6_17249	kinesin heavy	NS	0.85763263
s6_7574	achain structure of the complex of a mitotic kinesin with its calcium binding regulator	NS	0.84622635
s6_6486	kinesin heavy chain	NS	0.83623412
s6_28809	kinesin-like protein	NS	0.81400056
s6_8624	kinesin-like protein ncd	NS	0.80639429
s6_7735	kinesin-like protein	NS	0.79511854
s6_6956	achain structure of the complex of a mitotic kinesin with its calcium binding regulator	NS	0.78545555
s6_5750	kinesin heavy	NS	0.77548534
s6_1021	kinesin-like protein kifc1	NS	0.77231009
s6_2757	hypothetical protein GUITHDRAFT_65383, parti	NS	0.77223085
s6_11142	kinesin-like protein kif13a	NS	0.76411994
s6_12506	dynein heavy chain family protein	NS	0.73995654
s6_1510	kinesin-like protein	NS	0.73237914
s6_33482	kinesin family member 13a	NS	0.72401661

s6_29545	kinesin-like protein	NS	0.71849206
s6_16159	dynein heavy	NS	0.71626154
s6_40266	kinesin-ii motor subunit	NS	0.71311538
s6_22098	by-2 kinesin-like protein 5	NS	0.69375758
s6_311	kif3a protein	NS	0.64667245
s6_12552	kinesin motor domain-containing protein	NS	0.63062593
s6_27916	kinesin family member 3	NS	0.62871216
s6_16977	kinesin-ii subunit	NS	0.62550222
s6_12363	hypothetical protein GUITHDRAFT_106577	NS	0.61833066
s6_13022	kinesin-ii 95 kda	NS	0.60094858
s6_1226	dsk1_cylfu ame: full=diatom spindle kinesin 1	NS	0.59586991
s6_11611	kinesin motor expressed	NS	0.58159753
s6_4022	kinesin-like calmodulin binding protein	NS	0.57641068
s6_28632	kinesin-like protein	NS	0.56660715
s6_34173	kinesin family member 13bb	NS	0.4668263
s6_10697	kinesin-like protein 2-like	NS	0.44680842
s6_416	tubulin beta	NS	0.43621556
s6_51649	kinesin motor domain containing protein	NS	0.39695874
s6_35352	kinesin family-like protein	NS	0.3948966
s6_51007	kinesin motor domain protein	NS	0.35940602
s6_34057	kinesin-like protein	NS	0.33262884
s6_12770	kinesin-ii 85 kda	NS	0.3237186
s6_55327	alpha tubulin	NS	-0.3772378
s6_3464	intraflagellar transport protein 46 homolog	NS	-0.3807395
s6_38244	beta-tubulin	NS	-0.5130287
s6_39204	Tubulin beta chain	NS	-0.5503184
s6_4050	dynein heavy chain 11	NS	-0.5962254
s6_2471	beta-tubulin	NS	-0.6541101
s6_5313	beta-tubulin	NS	-0.7033248
s6_17057	kinesin delta 560-624	NS	-0.707916
s6_39108	ciliary dynein heavy	NS	-0.7116714
s6_19598	tubulin alpha chain	NS	-0.7623146
s6_34735	tubulin alpha chain	NS	-0.7880918
s6_5946	kinesin family member 13ba	NS	-0.8225844
s6_27934	tubulin alpha chain	NS	-0.8325367
s6_27837	tubulin alpha chain	NS	-0.8408163
s6_58664	alpha tubulin	NS	-0.9876181
s6_59556	alpha tubulin	NS	-1.0379471
s6_22789	Tubulin beta chain	NS	-1.1629517
s6_415	beta-tubulin	NS	-1.2559454
s6_56240	alpha- partial	NS	-1.4585106

NS= not significant
